# In Silico Prediction of Stratum Corneum Partition Coefficients via COSMOmic and Molecular Dynamics Simulations

**DOI:** 10.1021/acs.jpcb.2c08566

**Published:** 2023-03-17

**Authors:** Nicola Piasentin, Guoping Lian, Qiong Cai

**Affiliations:** † Department of Chemical and Process Engineering, University of Surrey, Guildford GU27XH, U.K.; ‡ Unilever R&D Colworth, Unilever, Sharnbrook MK441LQ, U.K.

## Abstract

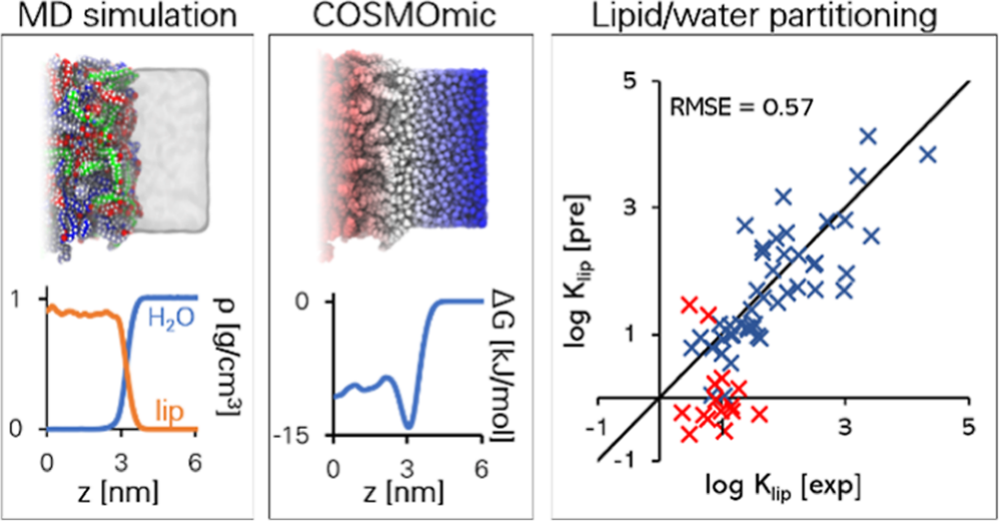

Stratum corneum (SC) is the main barrier of human skin where the inter-corneocytes lipids provide the main pathway for transdermal permeation of functional actives of skin care and health. Molecular dynamics (MD) has been increasingly used to simulate the SC lipid bilayer structure so that the barrier property and its affecting factors can be elucidated. Among reported MD simulation studies, solute partition in the SC lipids, an important parameter affecting SC permeability, has received limited attention. In this work, we combine MD simulation with COSMOmic to predict the partition coefficients of dermatologically relevant solutes in SC lipid bilayer. Firstly, we run MD simulations to obtain equilibrated SC lipid bilayers with different lipid types, compositions, and structures. Then, the simulated SC lipid bilayer structures are fed to COSMOmic to calculate the partition coefficients of the solutes. The results show that lipid types and bilayer geometries play a minor role in the predicted partition coefficients. For the more lipophilic solutes, the predicted results of solute partition in SC lipid bilayers agree well with reported experimental values of solute partition in extracted SC lipids. For the more hydrophilic molecules, there is a systematical underprediction. Nevertheless, the MD/COSMOmic approach correctly reproduces the phenomenological correlation between the SC lipid/water partition coefficients and the octanol/water partition coefficients. Overall, the results show that the MD/COSMOmic approach is a fast and valid method for predicting solute partitioning into SC lipids and hence supporting the assessment of percutaneous absorption of skin care ingredients, dermatological drugs as well as environmental pollutants.

## Introduction

The outermost layer of the epidermis, the stratum corneum (SC), acts as the main barrier in skin, avoiding excessive dehydration and shielding the body from external stresses.^1,2^ Percutaneous absorption takes place when the skin is exposed to soluble substances. Experimental evidence suggests that the inter-corneocytes lipid matrix provides the main pathway of percutaneous absorption.^2−7^ Current state-of-the-art considers the SC lipids as mainly composed of three lipid types, ceramides, free-fatty acids, and cholesterol, in roughly equimolar ratio.^8,9^

Understanding the structure and barrier property of the SC lipids is critically important for assessing percutaneous absorption, bioavailability, and safety of topically exposed chemicals, including cosmetics ingredients, dermatological drugs as well as environmental pollutants, to name few. Recently, molecular dynamics (MD) simulations have been exploited to simulate the structure and barrier properties of the SC lipid bilayers. Stemming from a united-atom parametrization of phospholipids,^10,11^ cholesterol,^12^ and free-fatty acids,^12^ early MD simulations^13−16^ considered the “classic” lipid bilayer structure, which presented ceramides in a hairpin configuration with their heads facing a water phase. Since then, several other structures have been investigated in different force field representations, spanning from stacked classic bilayers,^17,18^ to more complex gel-like systems with splayed ceramides and varying amount of water.^19−22^ Large coarse-grained models have been also reported^23−25^ and a few studies simulated the effect of topical molecules on lipids, for example, DMSO,^16^ ethanol,^26^ and others.^20,27^ Reported MD simulation studies of SC lipid bilayer permeability are limited to water,^14,17,19^ ethanol,^21^ and few others.^20,28,29^ Solute partition in SC lipids is an important parameter affecting SC permeability. Despite an increasing number of reported MD simulation studies, the partition of solutes to SC lipids has received less attention. A main challenge in the MD prediction of partition (as well as permeability) coefficients of SC lipids is that, despite the growth in computational power and the development of enhanced sampling methods,^28,30^ the sampling of Δ*G* for a single solute requires several microseconds of simulated dynamics of the system,^27^ which can easily reach hours or days of computational run on specialized high performance computer clusters.

A different approach for the prediction of partition coefficients (and other thermodynamical properties) is based on the so-called *conductor-like screening model for real solvents*, or COSMO-RS theory.^31−33^ The first step involves calculating via quantum mechanics, specifically density functional theory (DFT), the surface charge energy distribution of the molecular components of the system. The properties of interest for homogeneous systems are then derived via thermodynamic calculations of solute–solvent molecular surface interactions. The theory is implemented in the software COSMOtherm^34,35^ and has been recently generalized to non-homogeneous systems in COSMOmic.^36^ In this approach, the input system is subdivided along some symmetry axis into slices small enough to be considered homogeneous. The atomistic structures fed to COSMOmic can be obtained from MD simulations. This combined MD/COSMOmic has been recently used in several publications to successfully predict the partition coefficients of solutes in octanol/water biphasic systems,^37^ phospholipid bilayers,^38,39^ microemulsions,^40,41^ and micellar systems.^41−44^

COSMOmic is several orders of magnitude faster than MD simulations.^36^ The combined MD/COSMOmic approach requires the MD derived lipid bilayer assembly to be simulated only once, while in the full MD approach the sampling routine must be performed for every solute investigated.

In this work, we report progress in applying the combined MD/COSMOmic approach to predict the SC lipid/water partition coefficients *K*_lip_ for a set of dermatological relevant solutes. The intention is to investigate a new and fast method to predict *K*_lip_ and support in silico assessment of dermal delivery, bioavailability, and safety of wide topically exposed chemicals of cosmetics ingredients, dermatological drugs, as well as environmental pollutants. To this end, MD simulations have been conducted to generate several SC lipid system geometries of different lipid types and compositions that are then fed to COSMOmic to calculate the corresponding partition coefficients. The set of solutes consists of an experimental dataset of sixty-two molecules collected from literature. We then compare the predicted SC lipid/water partition coefficients with the experimental data to both assess the validity of the MD/COSMOmic approach and to examine the effect of the different simulated lipid bilayer geometries. Finally, in analogy to a quantitative structure–property relationship (QSPR) model reported in literature, we analyze the correlation between the predicted K_lip_ against both experimental and COSMOmic-predicted octanol/water partition coefficients to further benchmark and investigate the validity of the obtained results.

## Methods

### Lipid Bilayer System

**Figure 1:**
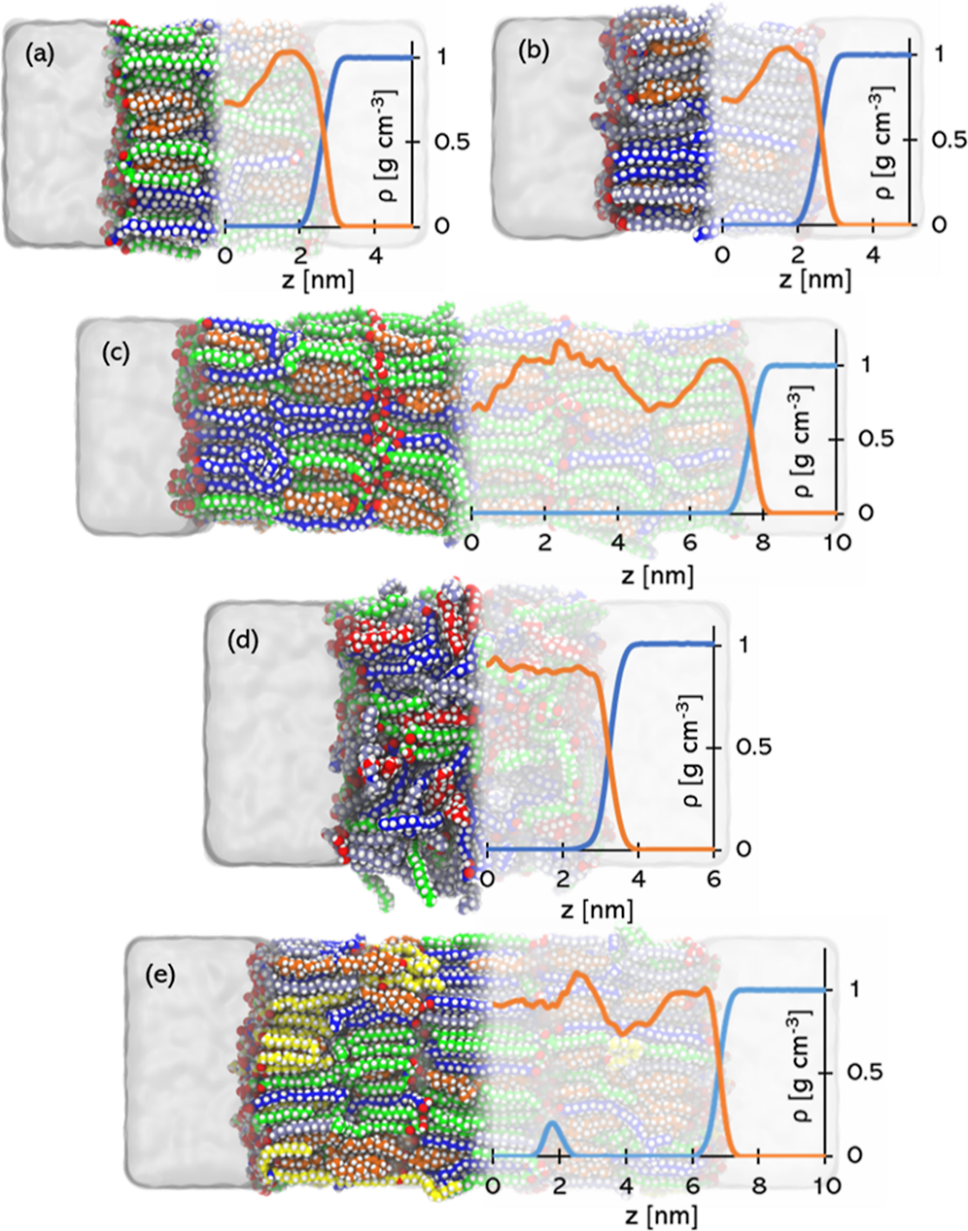
Snapshots of the systems studied and corresponding mass density profiles ρ. The systems are: (a) HPm2, (b) HPm3, (c) HPm2t, (d) Amor, and (e) LPP. Lipid atoms are represented via their vdW radii. Water is rendered as a white transparent volume. Lipid carbon atoms are blue (LIGN), orange (CHOL), green (CER2), grey (CER3), and yellow (CERO). Hydrogen, oxygen, and nitrogen atoms are white, red, and cyan, respectively. Mass densities of lipids (orange line) and water (cyan line) are symmetrised with respect to the bilayer centre.

The investigated SC lipid bilayer systems vary in geometry and lipid composition. The lipid types include lignoceric acid (LIGN), cholesterol (CHOL), and three types of ceramides of different length and conformation, that is, CER [NS] C24:0/18:1 (CER2), ceramide CER [NP] C24:0/18:0 (CER3), and the omega-*o*-acylceramide CER [EOS] (CERO). Five SC lipid systems have been built, which are shown in Figure 1. Their lipid composition and water content are summarized in Table 1. All systems have a mixed lipid composition with a 1:1:1 molar ratio of free fatty acids, cholesterol, and ceramides, as suggested by experimental evidence.^8,9^

**Table 1:** Number of Components for Each System Studied

	HPm2	HPm3	HPm2t	Amor	LPP
**CER2**	50	0	150	50	150
**CER3**	0	50	0	50	50
**CERO**	0	0	0	0	40
**LIGN**	50	50	150	100	240
**CHOL**	50	50	150	100	240
**H**_**2**_**O**	4500	4500	4500	9346	11235

Systems with ceramides only in hairpin conformation are named with the prefix *HPm*, followed by a number, specifying the content of CER2 (2) or CER3 (3). Therefore, HPm2 and HPm3 are a system with hairpin CER2 and a system with hairpin CER3, respectively. These two systems consist of a single bilayer with a water phase on both head sides, as shown in Figure 1a,b. The water content is of 30 water molecules per lipid, a configuration known as *fully-hydrated*, which has been extensively used in literature.^13,14,17,29,45^

Similarly, HPm2t, in Figure 1c, consists of three stacked mixed bilayers with hairpin CER2 (HPm2). Water molecules are placed only at the boundary head regions of the outermost leaflets, while the inner leaflets are in an anhydrous condition.

Figure 1d shows an amorphous system (*Amor*), which contains both CER2 and CER3 ceramides. The rationale behind this structure is that in experimental studies the lipids are extracted from the SC and may therefore lose the in-vivo structure in favor of an amorphous gel phase.

The last investigated structure, shown in Figure 1e, is a recently proposed model of the tri-layer long periodicity phase^46,47^ (LPP) of the SC lipid matrix.

### MD Simulations

All MD simulations are carried out in GROMACS2021.^48^ The CHARMM36 forcefield is used for lipid molecules.^49,50^ CHARMM TIP3P^51^ parameters are used for water.

The systems composed of a single lipid bilayer with hairpin conformation of ceramides, namely HPm2 and HPm3, are built in CHARMM-GUI.^52^ The equilibration runs are automatically generated by the web tool. They consist of an energy minimization via a steepest descent algorithm and a series of short (2.5–5 ns) *NVT* and *NPT* runs where the position restraints on lipids are gradually turned off.

The stacked bilayer system with also hairpin ceramide conformation, HPm2t, is based on the HPm2 system. The configuration is built by taking the equilibrated HPm2, stripping it from the water molecules, and replicating it three times along the *z* axis. Water molecules are then added back at the head region of the two outermost leaflets. After stacking the bilayers and adding the outer slabs of water, the system undergoes similar equilibration runs as the systems built via CHARMM-GUI reported in the latter. In the first equilibration stages, the compressibility on the x and y directions is set to zero to let the system relax along *z* and avoid lateral deformations of the leaflets.

The amorphous (Amor) system is built in-house by preparing two separate simulation boxes of the same dimension. In one, the lipid components are randomly placed at the given molar ratio. The other is a box of water. The boxes undergo *NVT* and *NPT* equilibrations, before being joined to form a biphasic lipid/water system. When separated, the compressibility along *x* and *y* is set to zero so that the boxes retain the same length, making it possible to directly join them at the end of the simulations. After the merging, a further 5 ns *NPT* equilibration simulation is performed with full pressure coupling.

The LPP model is taken from previous literature,^21^ to which the reader is referred for further details. The system (*model 1* of the cited paper) was kindly made available by the authors. Here it is modified by increasing the perimetral water content to better define a bulk water phase for COSMOmic calculations. No changes are made to the water content within the lipid phase, or to the lipid phase itself. Since the provided snapshot is already fully equilibrated, the equilibration phases consist only of short (3 ns) *NVT* and *NPT* runs with weak position restraints on the lipids to thermalize and relax the added water molecules.

For all the systems, after the equilibration runs an *NPT* production run of at least 50 ns is executed. A snapshot of the positions of the atoms is printed every 2500 steps (5 ps). The analysis presented in the following are performed on the last 10 ns of the production runs.

3D periodic boundary conditions are used. The time step is 2 fs. Electrostatic and van der Waals interactions are calculated via the PME method^53^ with a cut-off distance of 1.2 nm and a smooth force-switch from 1.0 to 1.2 nm for van der Waals interactions. The energy and pressure long-range dispersion corrections are off. H-bonds are constrained using the LINCS algorithm.^54^ Temperature is set to *T* = 303.15 K and is controlled via a Berendsen thermostat^55^ with a time constant of 0.2 ps during equilibration runs. It is then changed to the Bussi–Donadio–Parrinello v-rescale thermostat^56,57^ for production runs with a time constant of 2.0 ps. Lipids and water are coupled separately to the thermostat. Pressure is controlled by a semi-isotropic Berendsen barostat with time constant of 1 ps during equilibration and then by a Parrinello–Rahman semi-isotropic barostat^58^ with time constant of 5 ps during production run. For both barostats, compressibility is set to 4.5 × 10^–5^ bar^–1^ and pressure to 1 atm.

### COSMOmic Simulations

Lipid bilayer assemblies from MD simulations are extracted and set as the so-called .mic files for COSMOtherm input. They contain the structural information of the lipid bilayer assembly and the DFT calculation results of lipids and water molecules. In COSMOmic, the input structure is split along the *z* axis in slices thin enough to be considered homogenous. The free energy profile Δ*G* of each slice is calculated and integrated to provide the corresponding predicted partition coefficient. Previous works^38,39,42^ showed that slices of ca. 1 Å are sufficient for smooth and detailed energy profiles.

Each slice is populated with the (normalized) number distribution function of the atoms of the different molecules of the system. The procedure is automatically executed by COSMOmic once a structure file is provided. However, the provided snapshot cannot characterize the dynamic nature of the lipid leaflets, producing non-smooth probability densities and, consequently, non-smooth free energy profiles. This is overcome by calculating the normalized number densities over a time interval.^38,39^

The .mic file contains the DFT results for each lipid type in the bilayer. Since each lipid molecule at each time step is a different conformer of that lipid type, this raises the question of which conformer should be chosen to represent the lipid molecules in the given system. It has been shown^38,39^ that a good choice is picking a conformer with the average solvent-accessible surface area (SASA) for each lipid type within that system.

Here, the number of slices in the systems is chosen so that they are ca. 1 Å each. The normalized number density of each atom for all lipid types and the SASA distributions are calculated from the last 10 ns of the production runs with the GROMACS tools *gmx density* and *gmx sasa*, respectively. In *gmx sasa*, the probe dimension is that (automatic) of a water molecule. The density is then compiled in the COSMOmic format and symmetrized via an in-house python code. The conformer with the nearest to the average SASA is selected and extracted for DFT calculations.

The COSMO database of solutes consists of the sixty-four molecules for which measured experimental *K*_lip_ values were reported and compiled in Wang et al.^59^ (and references therein) and Ellison et al.^60^ The number of repeats for each measurement depends on the specific work and solute, but usually spaces from three, for example, progesterone and naphthol in Johnson et al.,^61^ to twenty-four, for example, for the measurements in Ellison et al.^60^ Thin layer chromatography measurements on extracted human SC lipids^62^ show ceramides/cholesterol/free fatty acids ratios spanning from 1:1:1 to 2:1:1, depending on body site and donor. The equimolar ratio adopted in this work is thus coherent with the expected composition of the extracted inter-corneocyte SC lipid phase.

The chemical structure of the solutes is downloaded from PubChem.^63^

DFT calculations are run in TmoleX^64^ (v21.0.0), which in turn relies on TURBOMOLE^65^ (v7.5) and its extension for COSMO-RS cavity surfaces.^66^ The calculations use the Becke–Perdew (BP) functional^67,68^ with the triple-ζ valence polarization (TZVP) basis set^69,70^ and the resolution of identity (RI) approximation.^71^ For the lipid and water molecules extracted from the simulated systems, single point DFT calculations are performed. For solutes, a conformer search is run with COSMOconf^72^ (v21.0), followed by a full geometry optimization and DFT calculation. The results are compiled into a COSMO database.

The calculation of solutes free energy profiles is performed in COSMOmic,^36^ which is implemented within the COSMOtherm^34^ (v21.0) software, with the COSMO-RS^32^ parameters set *BP_TZVP_21.ctd*. The free energy profiles are exported and integrated via an in-house python code.

A summary of the workflow, the SASA average values, and the database of solutes are collected in the Supporting Information.

### Partition Coefficient

The lipid/water partition coefficient *K*_lip_ of a solute is directly proportional to the Boltzmann weighted free energy. More than one formula has been proposed to calculate *K*_lip_.^39^ Here, the method proposed by Klamt et al.^36^ is used, which takes into account the full free energy profile and is stable also for strongly hydrophilic solutes. The lipid/water partition coefficient is expressed as
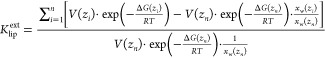
1where *n* is the total number of slices of the system, *V*(*z*_*i*_) is the volume of the *i*th slice, Δ*G*(*z*_*i*_) is the free energy at *z*_*i*_, *R* is the universal gas constant, *T* is the absolute temperature, and *x*_w_(*z*_*i*_) = *n*_w_(*z*_*i*_)/*n*_w,tot_ is the ratio of *n*_w_(*z*_*i*_), the number of water molecules contained in the *i*th slice, to *n*_w,tot_, the total number of water molecules in the system.

Equation 1 is an extensive quantity with units of mol/mol. It can be converted in the following intensive coefficient with the units of moles of solute per kg of lipid over moles of solute per liter of water [L/kg]
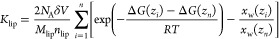
2where *N*_A_ is the Avogadro number, δ*V* is the volume of a single volume slice, *n*_lip_ is the total number of lipids, and *M*_lip_ is the molecular weight of the lipids. As stated, eq 2 implicitly requires that all system slices have the same volume δ*V*, condition met in the simulations conducted in this work. Its exact derivation is discussed in the Supporting Information.

### Visualization and Data Analysis

The bilayer structures are visualized with VMD^73^ and images rendered via Tachyon.^74^ Data analysis of the bilayer structure is carried out with the GROMACS tools *gmx density* and *gmx sasa*. Plots are fitted and produced in python3 via lmfit^75^ and matplotlib,^76^ respectively.

Both the root-mean-square error (RMSE) and the residuals (RES) between predicted and measured SC lipid/water partition coefficients are calculated. The RMSE is defined as the root square of the average of the squared difference between predicted and experimental values, that is
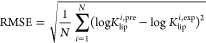
3where log *K*_lip_^*i*,pre^ (log *K*_lip_^*i*,exp^) is the base-ten logarithm of the predicted (experimental) lipid to water partition coefficient of the *i*th molecule in the dataset. Similarly, the residual of a solute is the difference between the predicted and the experimental value, that is
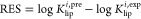
4

## Results and Discussions

**Figure 2:**
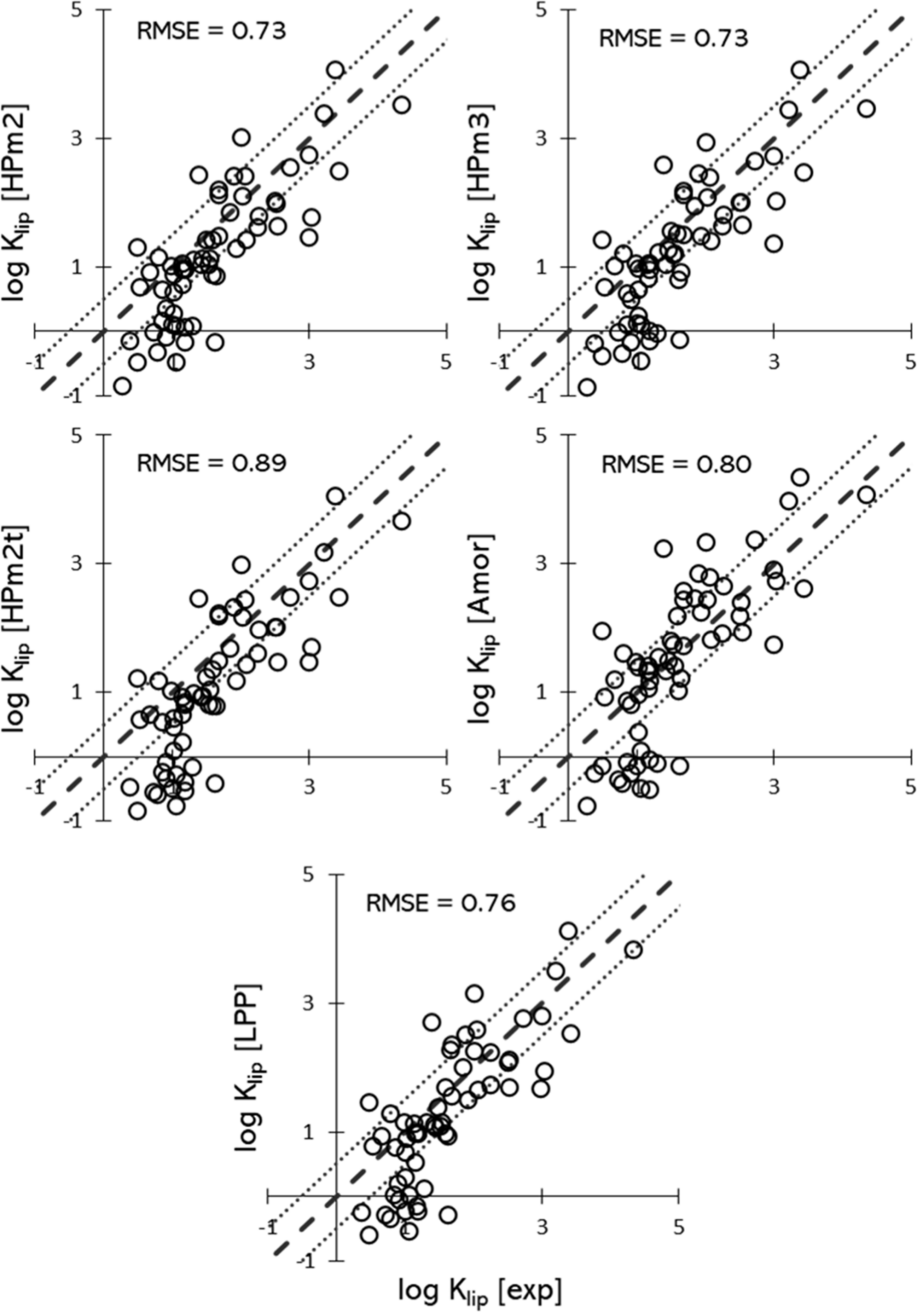
Predicted log *K*_lip_ plotted against the experimental ones for all the systems investigated. The thick dashed line indicates perfect agreement, the two thin dotted lines highlight the ±0.5 log point interval.

Figure 2 shows the predicted log *K*_lip_ plotted against the corresponding experimental values for the sixty-two solutes in all the investigated systems. The corresponding RMSE is also reported. Here and in all the following analysis, two molecules have been removed from the original dataset with sixty-four solutes, namely flufenamic acid and hydrocortisone octanoate, since it has been argued that for extremely lipophilic (log *K*_ow_ > 5.0) solutes the corresponding measured log *K*_lip_ depends on the lipid content of the sample.^77^

The results show that the single bilayer HPm2 and HPm3 systems have the lowest RMSE values of 0.73 and HPm2t has the highest RMSE value of 0.89. This shows that the prediction from the simple hairpin systems (HPm2 and HPm3) is better than the ones for the more complex systems.

The overall trend for the higher predicted log *K*_lip_ values is well represented, with most datapoints falling within the ±0.5 log points interval around the perfect agreement line. However, the partitioning values for the lower log *K*_lip_ interval are systematically underpredicted in all systems. This is clearly visible in all the plots of Figure 2 for predicted values of log *K*_lip_ < 0.5.

**Figure 3:**
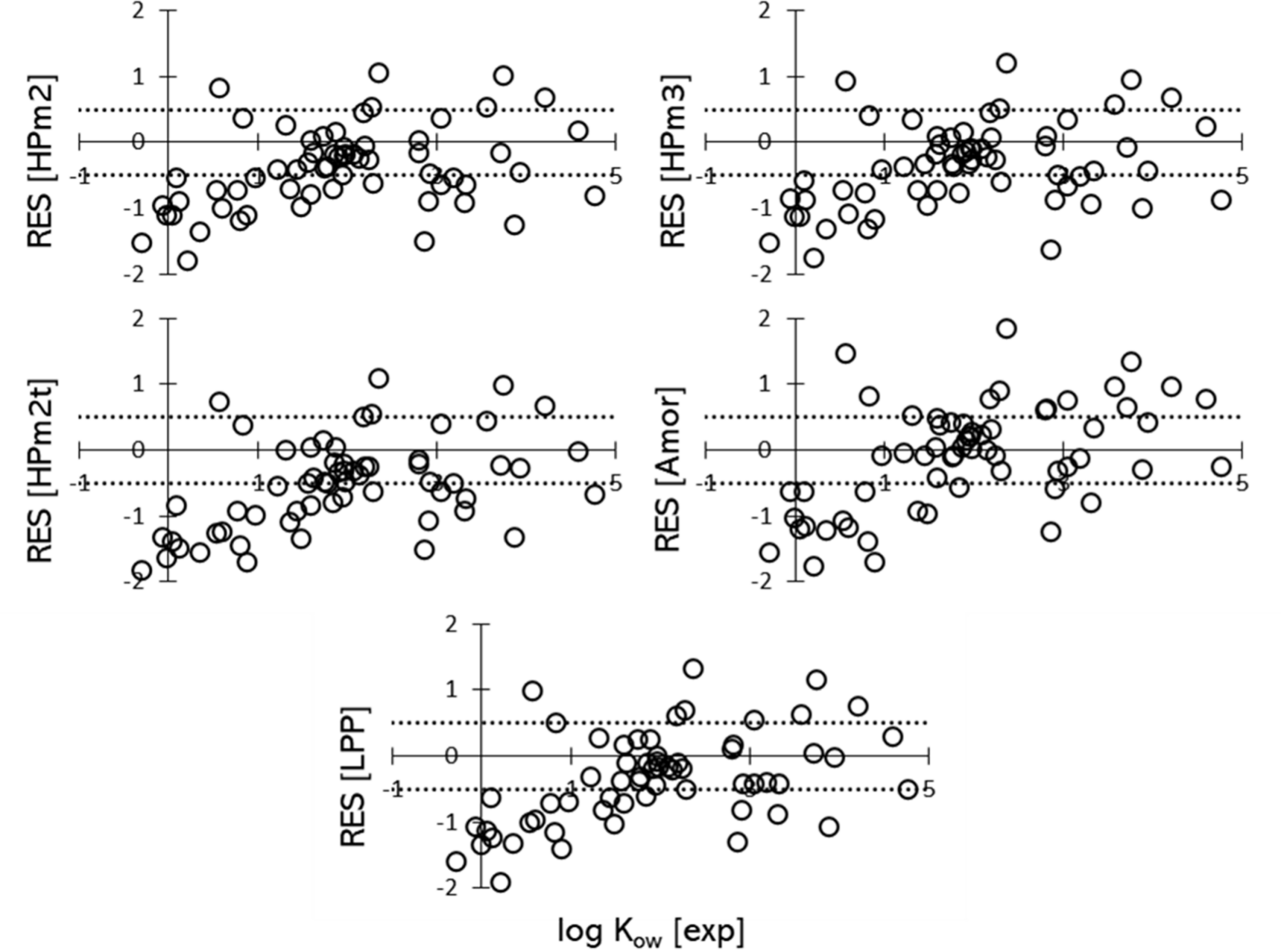
Residuals of log *K*_lip_ plotted against the experimental log *K*_ow_ coefficients for all the systems investigated. The two thin dotted lines highlight the ±0.5 log point interval.

Figure 3 shows the residues of the predicted log *K*_lip_, as defined in eq 4. In all cases, the residuals for solutes with log *K*_ow_ < 1 are mostly negative, indicating a tendency of the MD/COSMOmic approach to underestimate the predicted *K*_lip_ for the less lipophilic molecules. The absence of water within the lipid leaflets in most of the simulated systems could explain such systematic underprediction. Nevertheless, even the predicted log *K*_lip_ values in the LPP system, which is the only simulated structure that has a noticeable number of water molecules within the lipid structure (Figure 1), are still underestimated.

To further investigate this disagreement, the molecules with log *K*_ow_ < 1 have been removed and the RMSE values have been recalculated with the reduced datasets. Sixteen solutes are in this category, and their removal noticeably increases the RMSEs of the predictions, which are collected in Table 2. Most of these solutes are small (MW < 200 Da) and have a predicted log *K*_lip_ < 0.5, as can be seen in Figure S8 and Table S2. Notably, many of the hydrophilic solutes are amines, which are known to be problematic in DFT calculations due to the lone pair in the nitrogen atom in amine residues.^78^

**Table 2:** RMSE Values for all the Systems Investigated for the Whole Dataset (*All*) and for the Reduced Dataset (With Experimental log *K*_ow_ ≥ 1, *Reduced*)

		HPm2	HPm3	HPm2t	Amor	LPP
RMSE	all	0.73	0.73	0.89	0.80	0.76
	reduced	0.58	0.57	0.65	0.62	0.57

The partitioning of solutes into the SC lipids is usually correlated to the octanol/water partition *K*_ow_ via the following QSPR 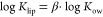
5with reported fitted values of β = 0.69,^59^ 0.70,^79,80^ 0.86,^81^ generally indicating that SC lipids are more hydrophilic than octanol.

Since the relationship in eq 5 is well aligned with the experimental data, it is worth investigating whether the same trend can be reproduced by the MD/COSMOmic approach. To this end, all the experimental log *K*_lip_ values have been compiled into a single database and fitted to eq 5. The octanol/water partition coefficients *K*_ow_ are taken from the EPA dashboard,^82^ which in turn relies on OPERA^83^ to generate in-silico predictions if no experimental log *K*_ow_ data is available for a given chemical. The best fitted relationship, summarized in the Supporting Information, has linear coefficient β = 0.74, RMSE = 0.46, and correlation coefficient *R* = 0.84.

**Figure 4:**
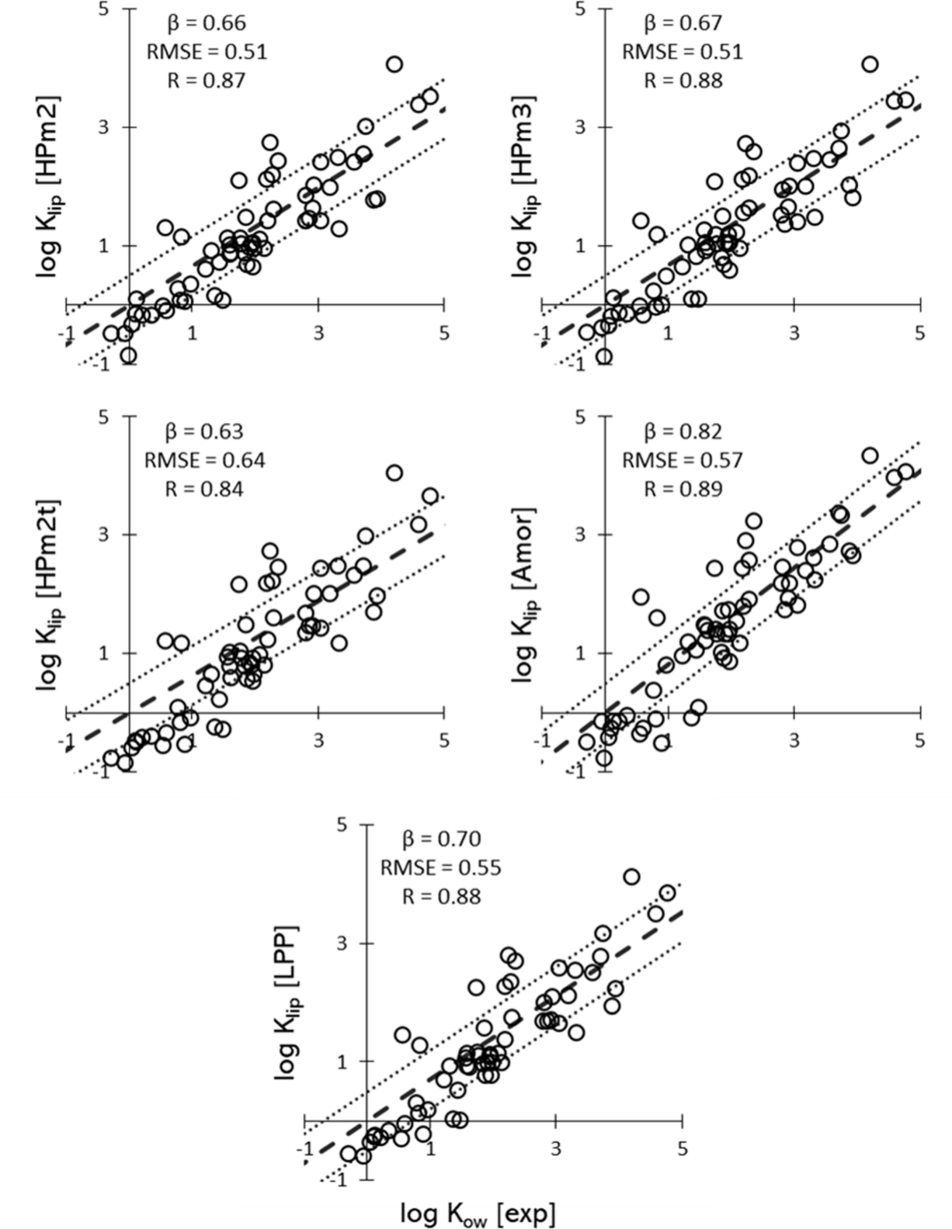
Predicted log *K*_lip_ plotted against the experimental log *K*_ow_ for all the systems investigated. The thick dashed line indicates perfect agreement, the two thin dotted lines highlight the ±0.5 log point interval.

Figure 4 shows the predicted log *K*_lip_ plotted against the log *K*_ow_ of the corresponding solutes. The datasets are fitted against the relationship in eq 5. The best fit results for the angular coefficient β, the RMSE, and the correlation coefficient R are reported alongside the corresponding subplot in Figure 4.

The results show that the experimentally observed proportionality between the lipophilicity of solutes and their ability to partition within SC lipids is well reproduced by the MD/COSMOmic approach. The predicted angular coefficients span the range 0.66 (HPm2)–0.82 (Amor), coherently with the value of β obtained from the fit of the experimental dataset (0.74). Figure 4 also supports the finding that, for hydrophilic molecules, log *K*_lip_ is systematically underpredicted since the near totality of datapoints with log *K*_ow_ < 1 falls below the perfect agreement curve.

**Figure 5:**
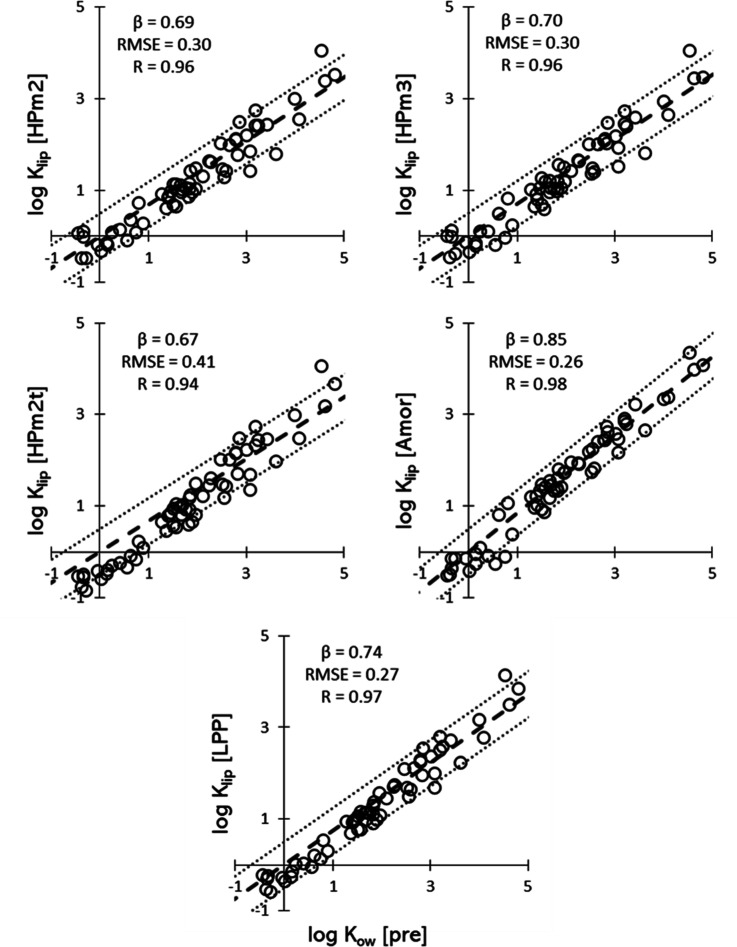
Predicted log *K*_lip_ plotted against the predicted log *K*_ow_ for all the systems investigated. The thick dashed line indicates perfect agreement, the two thin dotted lines highlight the ±0.5 log point interval.

COSMOtherm can also predict the log *K*_ow_ of solutes.^34,35^Figure 5 shows the predicted log *K*_lip_ plotted against COSMOtherm predicted log *K*_ow_. The datasets have been fitted against the relationship in eq 5, and the best fit results are again reported alongside the corresponding subplot in Figure 5.

In this case, the predicted angular coefficients span the range 0.67 (HPm2t)–0.85 (Amor), which is compatible with both the results in the latter and the angular coefficient obtained by fitting the experimental dataset. In all cases, β and *R* increase, while the RMSE decreases (see also Figure S12), indicating that the agreement with the linear approximation is stronger for the case where both coefficients are predicted. Here, the underprediction for log *K*_ow_ < 1 is much less pronounced, with the datapoints closer to the perfect agreement line in that axis region. This makes so that the linear fit has generally a larger β with respect to the fit against experimental log *K*_ow_, resulting in more lipophilic lipid bilayers.

Residuals of predicted log *K*_lip_ and log *K*_ow_ show some degree of correlation, as can be seen from Figures S10 and S11, which report the residuals between the predicted log *K*_lip_ datasets as a function of the residuals between COSMOtherm predicted and EPA log *K*_ow_ values. The first and the third quadrant are more populated than the other two, meaning that to an over(under)prediction of log *K*_ow_ corresponds an over(under)prediction of log *K*_lip_. Conversely, the residuals between predicted *K*_lip_ values and the linear relationship fitted from predicted *K*_ow_ are more evenly distributed. This is to be expected since the systematic bias is lost by using both predicted log *K*_lip_ and log *K*_ow_ for the fit of the linear relationship in eq 5.

## Conclusions

In this work, the combined MD/COSMOmic method has been used to predict the partition coefficient between SC lipids and water for a set of dermatological relevant solutes. Some of the most common SC lipid structures proposed in MD literature have been simulated. Then, the resulting equilibrated structures have been extracted and imported to COSMOmic to predict SC lipid/water partition coefficients. The log *K*_lip_ coefficients are predicted with a RMSE ranging from 0.73 to 0.89. The MD/COSMOmic approach tends to systematically underpredict the partition coefficients of the least lipophilic solutes (log *K*_ow_ < 1). The rationale behind this underprediction is unclear. A reason could be the absence of water in the simulated lipid structures. However, the underprediction is also present in the LPP system, which contains more water molecules within the lipid phase.

The best results have been obtained for hairpin fully hydrated models, which are not truly representatives of the physical state of in-vivo SC lipid bilayer sheets. The experimental studies from which the data was collected, that is, Ellison et al.^60^ and Wang et al.^59^ (and references therein), follow the same experimental procedure to measure the partition coefficients *K*_lip_. The lipids are firstly extracted from the epidermis by using a chloroform/methanol solution and then they are exposed to a water solution containing the solute of interest. Eventually, *K*_lip_ is measured as the ratio of the concentration of solute partitioned into the lipids to the concentration of solute in the solution. All datapoints (exception made for one in Wang et al.) were collected from ex-vivo human SC lipids. Notably, the process of extraction may cause the loss of the in vivo SC lipid structures, which was the rationale behind the study of an amorphous system among the geometries investigated in this work. Most importantly, the datapoints were collected in different studies and with different conditions, for example, pH and temperature, or with solutions containing other compounds to aid solubility or avoid oxidation, for example, those in Ellison et al*.*^60^ In this light, the pure water phase exploited here is not a faithful representation of the experimental set-ups. Nevertheless, the linear QSPRs from the fit to experimental data and from the fit to the predicted coefficients are in good agreement, which means that, to a certain extent, the differences in the set-ups have minor impacts on the experimental data and on the values predicted by the MD/COSMOmic method. In addition, the fit of the predicted log *K*_lip_ against both the experimental and predicted log *K*_ow_ shows that, for all the simulated systems, SC lipids are less lipophilic than octanol, as also observed experimentally.^59,77^ In conclusion, the results obtained suggest that the MD/COSMOmic approach can correctly reproduce the characteristics of the partition properties of SC lipids.

## Supporting Information Available

Summary of the workflow; SASA and individual number density distributions; SASA average values; derivation of partition coefficient formula; fit of experimental data; residuals and fit analysis of predicted dataset (PDF)
Excel sheet with experimental and predicted datasets; individual free energy profiles for all the systems and all the solutes (XLSX)

## Abbreviations

CER: ceramide
CHOL: cholesterol
COSMO-RS: conductor-like screening model for real solvents
DFT: density functional theory
DMSO: dimethyl sulfoxide
LIGN: lignoceric acid
LPP: long periodicity phase
MD: molecular dynamics
QSPR: quantitative structure–property relationship
RES: residual
RMSE: root-mean-square error
SASA: solvent-accessible surface area
SC: stratum corneum

## References

[ref1] Elias P. M.; Feingold K. R. Skin barrier; CRC Press, 2005.

[ref2] Madison K. C. Barrier function of the skin: “la raison d’etre” of the epidermis. J. Invest. Dermatol. 2003, 121, 231–241. 10.1046/j.1523-1747.2003.12359.x. 12880413

[ref3] Berthaud F.; Boncheva M. Correlation between the properties of the lipid matrix and the degrees of integrity and cohesion in healthy human Stratum corneum. Exp. Dermatol. 2011, 20, 255–262. 10.1111/j.1600-0625.2010.01164.x. 21054560

[ref4] Feingold K. R.; Elias P. M. Role of lipids in the formation and maintenance of the cutaneous permeability barrier. Biochim. Biophys. Acta 2014, 1841, 280–294. 10.1016/j.bbalip.2013.11.007. 24262790

[ref5] Feingold K. R. Thematic review series: skin lipids. The role of epidermal lipids in cutaneous permeability barrier homeostasis. J. Lipid Res. 2007, 48, 2531–2546. 10.1194/jlr.R700013-JLR200. 17872588

[ref6] Elias P. M.; Friend D. S. The permeability barrier in mammalian epidermis. J. Cell Biol. 1975, 65, 180–191. 10.1083/jcb.65.1.180. 1127009 PMC2111161

[ref7] Elias P. M.; Menon G. K. Structural and lipid biochemical correlates of the epidermal permeability barrier. Adv. Lipid Res. 1991, 24, 1–26. 10.1016/b978-0-12-024924-4.50005-5. 1763710

[ref8] Wertz P. W.; van den Bergh B. The physical, chemical and functional properties of lipids in the skin and other biological barriers. Chem. Phys. Lipids 1998, 91, 85–96. 10.1016/s0009-3084(97)00108-4. 9569614

[ref9] McGrath J. A.; Eady R. A. J.; Pope F. M. Anatomy and organization of human skin. Rook’s textbook of dermatology; Wiley-Blackwell, 2004; pp 3.1–3.15.

[ref10] Chiu S.-W.; Clark M.; Balaji V.; Subramaniam S.; Scott H. L.; Jakobsson E. Incorporation of surface tension into molecular dynamics simulation of an interface: a fluid phase lipid bilayer membrane. Biophys. J. 1995, 69, 1230–1245. 10.1016/s0006-3495(95)80005-6. 8534794 PMC1236354

[ref11] Berger O.; Edholm O.; Jähnig F. Molecular dynamics simulations of a fluid bilayer of dipalmitoylphosphatidylcholine at full hydration, constant pressure, and constant temperature. Biophys. J. 1997, 72, 2002–2013. 10.1016/s0006-3495(97)78845-3. 9129804 PMC1184396

[ref12] Höltje M.; Förster T.; Brandt B.; Engels T.; von Rybinski W.; Höltje H.-D. Molecular dynamics simulations of stratum corneum lipid models: fatty acids and cholesterol. Biochim. Biophys. Acta, Biomembr. 2001, 1511, 156–167. 10.1016/s0005-2736(01)00270-x. 11248214

[ref13] Das C.; Noro M. G.; Olmsted P. D. Simulation studies of stratum corneum lipid mixtures. Biophys. J. 2009, 97, 1941–1951. 10.1016/j.bpj.2009.06.054. 19804725 PMC2756355

[ref14] Das C.; Olmsted P. D.; Noro M. G. Water permeation through stratum corneum lipid bilayers from atomistic simulations. Soft Matter 2009, 5, 4549–4555. 10.1039/b911257j.

[ref15] Notman R.; Anwar J.; Briels W. J.; Noro M. G.; den Otter W. K. Simulations of skin barrier function: free energies of hydrophobic and hydrophilic transmembrane pores in ceramide bilayers. Biophys. J. 2008, 95, 4763–4771. 10.1529/biophysj.108.138545. 18708461 PMC2576386

[ref16] Notman R.; den Otter W. K.; Noro M. G.; Briels W. J.; Anwar J. The permeability enhancing mechanism of DMSO in ceramide bilayers simulated by molecular dynamics. Biophys. J. 2007, 93, 2056–2068. 10.1529/biophysj.107.104703. 17513383 PMC1959535

[ref17] Del Regno A.; Notman R. Permeation pathways through lateral domains in model membranes of skin lipids. Phys. Chem. Chem. Phys. 2018, 20, 2162–2174. 10.1039/c7cp03258g. 29116267

[ref18] Wang E.; Klauda J. B. Structure and Permeability of Ceramide Bilayers and Multilayers. J. Phys. Chem. B 2019, 123, 2525–2535. 10.1021/acs.jpcb.9b00037. 30795646

[ref19] Lundborg M.; Narangifard A.; Wennberg C. L.; Lindahl E.; Daneholt B.; Norlén L. Human skin barrier structure and function analyzed by cryo-EM and molecular dynamics simulation. J. Struct. Biol. 2018, 203, 149–161. 10.1016/j.jsb.2018.04.005. 29702212

[ref20] Lundborg M.; Wennberg C. L.; Narangifard A.; Lindahl E.; Norlén L. Predicting drug permeability through skin using molecular dynamics simulation. J. Controlled Release 2018, 283, 269–279. 10.1016/j.jconrel.2018.05.026. 29864475

[ref21] Wang E.; Klauda J. B. Molecular structure of the long periodicity phase in the stratum corneum. J. Am. Chem. Soc. 2019, 141, 16930–16943. 10.1021/jacs.9b08995. 31547662

[ref22] Beddoes C. M.; Gooris G. S.; Foglia F.; Ahmadi D.; Barlow D. J.; Lawrence M. J.; Demé B.; Bouwstra J. A. Arrangement of ceramides in the skin: sphingosine chains localize at a single position in stratum corneum lipid matrix models. Langmuir 2020, 36, 10270–10278. 10.1021/acs.langmuir.0c01992. 32816488 PMC7498151

[ref23] Gupta R.; Rai B. Penetration of Gold Nanoparticles through Human Skin: Unraveling Its Mechanisms at the Molecular Scale. J. Phys. Chem. B 2016, 120, 7133–7142. 10.1021/acs.jpcb.6b03212. 27362257

[ref24] Gupta R.; Rai B. Effect of Size and Surface Charge of Gold Nanoparticles on their Skin Permeability: A Molecular Dynamics Study. Sci. Rep. 2017, 7, 45292 10.1038/srep45292. 28349970 PMC5368607

[ref25] Antunes E.; Cavaco-Paulo A. Stratum corneum lipid matrix with unusual packing: A molecular dynamics study. Colloids Surf., B 2020, 190, 110928 10.1016/j.colsurfb.2020.110928. 32179416

[ref26] Gupta R.; Badhe Y.; Rai B.; Mitragotri S. Molecular mechanism of the skin permeation enhancing effect of ethanol: a molecular dynamics study. RSC Adv. 2020, 10, 12234–12248. 10.1039/d0ra01692f. 35497613 PMC9050718

[ref27] Lundborg M.; Wennberg C.; Lidmar J.; Hess B.; Lindahl E.; Norlén L. Skin Permeability Prediction with MD Simulation Sampling Spatial and Alchemical Reaction Coordinates. Biophys. J. 2022, 121, 3837 10.1016/j.bpj.2022.09.009. 36104960 PMC9674988

[ref28] Lundborg M.; Wennberg C.; Lidmar J.; Hess B.; Lindahl E.; Norlén L. Predictions of Skin Permeability Using Molecular Dynamics Simulation from Two-Dimensional Sampling of Spatial and Alchemical Perturbation Reaction Coordinates. 2022, bioRxiv:479880.

[ref29] Gupta R.; Sridhar D. B.; Rai B. Molecular Dynamics Simulation Study of Permeation of Molecules through Skin Lipid Bilayer. J. Phys. Chem. B 2016, 120, 8987–8996. 10.1021/acs.jpcb.6b05451. 27518707

[ref30] Kosztin I.; Barz B.; Janosi L. Calculating potentials of mean force and diffusion coefficients from nonequilibrium processes without Jarzynski’s equality. J. Chem. Phys. 2006, 124, 064106 10.1063/1.2166379. 16483195

[ref31] Klamt A. Conductor-like screening model for real solvents: a new approach to the quantitative calculation of solvation phenomena. J. Phys. Chem. 1995, 99, 2224–2235. 10.1021/j100007a062.

[ref32] Klamt A.; Jonas V.; Bürger T.; Lohrenz J. C. Refinement and parametrization of COSMO-RS. J. Phys. Chem. A 1998, 102, 5074–5085. 10.1021/jp980017s.

[ref33] Eckert F.; Klamt A. Fast solvent screening via quantum chemistry: COSMO-RS approach. AIChE J. 2002, 48, 369–385. 10.1002/aic.690480220.

[ref34] Klamt A.; Eckert F. Prediction of vapor liquid equilibria using COSMOtherm. Fluid Phase Equil. 2004, 217, 53–57. 10.1016/j.fluid.2003.08.018.

[ref35] Klamt A.; Eckert F.; Diedenhofen M. Prediction or partition coefficients and activity coefficients of two branched compounds using COSMOtherm. Fluid Phase Equil. 2009, 285, 15–18. 10.1016/j.fluid.2009.05.010.

[ref36] Klamt A.; Huniar U.; Spycher S.; Keldenich J. r. COSMOmic: a mechanistic approach to the calculation of membrane– water partition coefficients and internal distributions within membranes and micelles. J. Phys. Chem. B 2008, 112, 12148–12157. 10.1021/jp801736k. 18754634

[ref37] Turchi M.; Cai Q.; Lian G. An evaluation of in-silico methods for predicting solute partition in multiphase complex fluids–A case study of octanol/water partition coefficient. Chem. Eng. Sci. 2019, 197, 150–158. 10.1016/j.ces.2018.12.003.

[ref38] Jakobtorweihen S.; Ingram T.; Smirnova I. Combination of COSMOmic and molecular dynamics simulations for the calculation of membrane–water partition coefficients. J. Comput. Chem. 2013, 34, 1332–1340. 10.1002/jcc.23262. 23447371

[ref39] Jakobtorweihen S.; Zuniga A. C.; Ingram T.; Gerlach T.; Keil F.; Smirnova I. Predicting solute partitioning in lipid bilayers: Free energies and partition coefficients from molecular dynamics simulations and COSMOmic. J. Chem. Phys. 2014, 141, 045102 10.1063/1.4890877. 25084963

[ref40] Turchi M.; Cai Q.; Lian G. In Silico Prediction of the Thermodynamic Equilibrium of Solute Partition in Multiphase Complex Fluids: A Case Study of Oil–Water Microemulsion. Langmuir 2019, 35, 10855–10865. 10.1021/acs.langmuir.9b01513. 31335154

[ref41] Turchi M.; Lian G.; Cai Q.; Wood I.; Rabone J.; Noro M. Multi-scale modelling of solute partition equilibria of micelle-water and microemulsion-water systems using molecular dynamics and COSMOtherm. Computer Aided Chemical Engineering; Elsevier, 2017; Vol. 40, pp 2773–2778.

[ref42] Ingram T.; Storm S.; Kloss L.; Mehling T.; Jakobtorweihen S.; Smirnova I. Prediction of micelle/water and liposome/water partition coefficients based on molecular dynamics simulations, COSMO-RS, and COSMOmic. Langmuir 2013, 29, 3527–3537. 10.1021/la305035b. 23398189

[ref43] Yordanova D.; Ritter E.; Gerlach T.; Jensen J.-H.; Smirnova I.; Jakobtorweihen S. Solute partitioning in micelles: Combining molecular dynamics simulations, COSMOmic, and experiments. J. Phys. Chem. B 2017, 121, 5794–5809. 10.1021/acs.jpcb.7b03147. 28534622

[ref44] Yordanova D.; Ritter E.; Smirnova I.; Jakobtorweihen S. Micellization and partition equilibria in mixed nonionic/ionic micellar systems: predictions with molecular models. Langmuir 2017, 33, 12306–12316. 10.1021/acs.langmuir.7b02813. 28967760

[ref45] Wang E.; Klauda J. B. Simulations of Pure Ceramide and Ternary Lipid Mixtures as Simple Interior Stratum Corneum Models. J. Phys. Chem. B 2018, 122, 2757–2768. 10.1021/acs.jpcb.8b00348. 29466860

[ref46] Bouwstra J.; Gooris G.; Ponec M. The lipid organisation of the skin barrier: liquid and crystalline domains coexist in lamellar phases. J. Biol. Phys. 2002, 28, 211–223. 10.1023/A:1019983715589. 23345770 PMC3456653

[ref47] Bouwstra J. A.; Honeywell-Nguyen P. L.; Gooris G. S.; Ponec M. Structure of the skin barrier and its modulation by vesicular formulations. Prog. Lipid Res. 2003, 42, 1–36. 10.1016/s0163-7827(02)00028-0. 12467638

[ref48] Van Der Spoel D.; Lindahl E.; Hess B.; Groenhof G.; Mark A. E.; Berendsen H. J. GROMACS: fast, flexible, and free. J. Comput. Chem. 2005, 26, 1701–1718. 10.1002/jcc.20291. 16211538

[ref49] Venable R. M.; Sodt A. J.; Rogaski B.; Rui H.; Hatcher E.; MacKerell A. D. Jr; Pastor R. W.; Klauda J. B. CHARMM all-atom additive force field for sphingomyelin: elucidation of hydrogen bonding and of positive curvature. Biophys. J. 2014, 107, 134–145. 10.1016/j.bpj.2014.05.034. 24988348 PMC4119286

[ref50] Klauda J. B.; Venable R. M.; Freites J. A.; O’Connor J. W.; Tobias D. J.; Mondragon-Ramirez C.; Vorobyov I.; MacKerell A. D. Jr.; Pastor R. W. Update of the CHARMM all-atom additive force field for lipids: validation on six lipid types. J. Phys. Chem. B 2010, 114, 7830–7843. 10.1021/jp101759q. 20496934 PMC2922408

[ref51] MacKerell A. D. Jr; Bashford D.; Bellott M.; Dunbrack R. L. Jr.; Evanseck J. D.; Field M. J.; Fischer S.; Gao J.; Guo H.; Ha S.; Joseph-McCarthy D.; Kuchnir L.; Kuczera K.; Lau F. T. K.; Mattos C.; Michnick S.; Ngo T.; Nguyen D. T.; Prodhom B.; Reiher W. E.; Roux B.; Schlenkrich M.; Smith J. C.; Stote R.; Straub J.; Watanabe M.; Wiórkiewicz-Kuczera J.; Yin D.; Karplus M. All-atom empirical potential for molecular modeling and dynamics studies of proteins. J. Phys. Chem. B 1998, 102, 3586–3616. 10.1021/jp973084f. 24889800

[ref52] Wu E. L.; Cheng X.; Jo S.; Rui H.; Song K. C.; Dávila-Contreras E. M.; Qi Y.; Lee J.; Monje-Galvan V.; Venable R. M.; et al. CHARMM-GUI Membrane Builder toward realistic biological membrane simulations. J. Comput. Chem. 2014, 35, 1997–2004. 10.1002/jcc.23702. 25130509 PMC4165794

[ref53] Darden T.; York D.; Pedersen L. Particle mesh Ewald: An N· log (N) method for Ewald sums in large systems. J. Chem. Phys. 1993, 98, 10089–10092. 10.1063/1.464397.

[ref54] Hess B.; Bekker H.; Berendsen H. J.; Fraaije J. G. LINCS: a linear constraint solver for molecular simulations. J. Comput. Chem. 1997, 18, 1463–1472. 10.1002/(sici)1096-987x(199709)18:12<1463::aid-jcc4>3.0.co;2-h.

[ref55] Berendsen H. J. C.; Postma J. P. M.; van Gunsteren W. F.; DiNola A.; Haak J. R. Molecular dynamics with coupling to an external bath. J. Chem. Phys. 1984, 81, 3684–3690. 10.1063/1.448118.

[ref56] Bussi G.; Donadio D.; Parrinello M. Canonical sampling through velocity rescaling. J. Chem. Phys. 2007, 126, 014101 10.1063/1.2408420. 17212484

[ref57] Bussi G.; Zykova-Timan T.; Parrinello M. Isothermal-isobaric molecular dynamics using stochastic velocity rescaling. J. Chem. Phys. 2009, 130, 074101 10.1063/1.3073889. 19239278

[ref58] Parrinello M.; Rahman A. Polymorphic transitions in single crystals: A new molecular dynamics method. J. Appl. Phys. 1981, 52, 7182–7190. 10.1063/1.328693.

[ref59] Wang L.; Chen L.; Lian G.; Han L. Determination of partition and binding properties of solutes to stratum corneum. Int. J. Pharm. 2010, 398, 114–122. 10.1016/j.ijpharm.2010.07.035. 20674724

[ref60] Ellison C. A.; Tankersley K. O.; Obringer C. M.; Carr G. J.; Manwaring J.; Rothe H.; Duplan H.; Géniès C.; Grégoire S.; Hewitt N. J.; Jamin C. J.; Klaric M.; Lange D.; Rolaki A.; Schepky A. Partition coefficient and diffusion coefficient determinations of 50 compounds in human intact skin, isolated skin layers and isolated stratum corneum lipids. Toxicol. in Vitro 2020, 69, 104990 10.1016/j.tiv.2020.104990. 32882340

[ref61] Johnson M. E.; Blankschtein D.; Langer R. Evaluation of solute permeation through the stratum corneum: lateral bilayer diffusion as the primary transport mechanism. J. Pharm. Sci. 1997, 86, 1162–1172. 10.1021/js960198e. 9344175

[ref62] Weerheim A.; Ponec M. Determination of stratum corneum lipid profile by tape stripping in combination with high-performance thin-layer chromatography. Arch. Dermatol. Res. 2001, 293, 191–199. 10.1007/s004030100212. 11380152

[ref63] Kim S.; Chen J.; Cheng T.; Gindulyte A.; He J.; He S.; Li Q.; Shoemaker B. A.; Thiessen P. A.; Yu B.; Zaslavsky L.; Zhang J.; Bolton E. E. PubChem in 2021: new data content and improved web interfaces. Nucleic Acids Res. 2021, 49, D1388–D1395. 10.1093/nar/gkaa971. 33151290 PMC7778930

[ref64] Steffen C.; Thomas K.; Huniar U.; Hellweg A.; Rubner O.; Schroer A. TmoleX—a graphical user interface for TURBOMOLE. J. Comput. Chem. 2010, 31, 2967–2970. 10.1002/jcc.21576. 20928852

[ref65] Ahlrichs R.; Bär M.; Häser M.; Horn H.; Kölmel C. Electronic structure calculations on workstation computers: The program system turbomole. Chem. Phys. Lett. 1989, 162, 165–169. 10.1016/0009-2614(89)85118-8.

[ref66] Schäfer A.; Klamt A.; Sattel D.; Lohrenz J. C.; Eckert F. COSMO Implementation in TURBOMOLE: Extension of an efficient quantum chemical code towards liquid systems. Phys. Chem. Chem. Phys. 2000, 2, 2187–2193. 10.1039/B000184H.

[ref67] Perdew J. P. Density-functional approximation for the correlation energy of the inhomogeneous electron gas. Phys. Rev. B 1986, 33, 8822 10.1103/physrevb.33.8822. 9938299

[ref68] Becke A. D. Density-functional exchange-energy approximation with correct asymptotic behavior. Phys. Rev. A: At., Mol., Opt. Phys. 1988, 38, 3098 10.1103/physreva.38.3098. 9900728

[ref69] Eichkorn K.; Treutler O.; Öhm H.; Häser M.; Ahlrichs R. Auxiliary basis sets to approximate Coulomb potentials. Chem. Phys. Lett. 1995, 240, 283–290. 10.1016/0009-2614(95)00621-a.

[ref70] Eichkorn K.; Weigend F.; Treutler O.; Ahlrichs R. Auxiliary basis sets for main row atoms and transition metals and their use to approximate Coulomb potentials. Theor. Chem. Acc. 1997, 97, 119–124. 10.1007/s002140050244.

[ref71] Schäfer A.; Huber C.; Ahlrichs R. Fully optimized contracted Gaussian basis sets of triple zeta valence quality for atoms Li to Kr. J. Chem. Phys. 1994, 100, 5829–5835. 10.1063/1.467146.

[ref72] Systèmes D. COSMOconf, Version 21.0. 0; Dassault Systèmes: Biovia, 2020.

[ref73] Humphrey W.; Dalke A.; Schulten K. VMD: visual molecular dynamics. J. Mol. Graph. 1996, 14, 33–38. 10.1016/0263-7855(96)00018-5. 8744570

[ref74] Li H.; Ghodsi A.; Zaharia M.; Shenker S.; Stoica I. Tachyon: Reliable, memory speed storage for cluster computing frameworks. Proceedings of the ACM Symposium on Cloud Computing, 2014; pp 1–15.

[ref75] Newville M.; Stensitzki T.; Allen D. B.; Rawlik M.; Ingargiola A.; Nelson A. LMFIT: Non-linear least-square minimization and curve-fitting for Python. Astrophysics Source Code Library, 2016, ascl: 1606.1014.

[ref76] Hunter J. D. Matplotlib: A 2D graphics environment. IEEE Ann. Hist. Comput. 2007, 9, 90–95. 10.1109/mcse.2007.55.

[ref77] Nitsche J. M.; Wang T.-F.; Kasting G. B. A two-phase analysis of solute partitioning into the stratum corneum. J. Pharmaceut. Sci. 2006, 95, 649–666. 10.1002/jps.20549. 16432875

[ref78] Eckert F.; Klamt A. Accurate prediction of basicity in aqueous solution with COSMO-RS. J. Comput. Chem. 2006, 27, 11–19. 10.1002/jcc.20309. 16235262

[ref79] Potts R. O.; Guy R. H. Predicting skin permeability. Pharm. Res. 1992, 09, 663–669. 10.1023/a:1015810312465. 1608900

[ref80] Mitragotri S. Modeling skin permeability to hydrophilic and hydrophobic solutes based on four permeation pathways. J. Controlled Release 2003, 86, 69–92. 10.1016/s0168-3659(02)00321-8. 12490374

[ref81] Johnson M. E. Biophysical aspects of transdermal drug delivery and chemical enhancement; Massachusetts Institute of Technology, 1996.

[ref82] Williams A. J.; Grulke C. M.; Edwards J.; McEachran A. D.; Mansouri K.; Baker N. C.; Patlewicz G.; Shah I.; Wambaugh J. F.; Judson R. S. The CompTox Chemistry Dashboard: a community data resource for environmental chemistry. J. Cheminf. 2017, 9, 61 10.1186/s13321-017-0247-6. PMC5705535 29185060

[ref83] Kamel M.; Grulke C. M.; Judson R. S.; Williams A. J. OPERA models for predicting physicochemical properties and environmental fate endpoints. J. Cheminf. 2018, 10, 10 10.1186/s13321-018-0263-1. PMC5843579 29520515

