# Supplementary material for: In Silico Prediction of Stratum Corneum Partition Coefficients via COSMOmic and Molecular Dynamics Simulations
Source: J Phys Chem B. 2023 Mar 17;127(12):2719–28. doi: 10.1021/acs.jpcb.2c08566 (PMC10068742; doi:10.1021/acs.jpcb.2c08566)
Supplement: Supplementary file 1 — jp2c08566_si_001.pdf [file jp2c08566_si_001.pdf]

In-Silico Prediction of Stratum Corneum Partition Coefficients via COSMOmic and Molecular Dynamics Simulations

*Nicola Piasentin [a,b], Guoping Lian * [a,b], Qiong Cai [a]*

[a] Department of Chemical and Process Engineering, University of Surrey, Guildford GU27XH, U.K.

[b] Unilever R&D Colworth, Unilever, Sharnbrook MK441LQ, U.K.

1. SASA and individual density analysis

Workflow

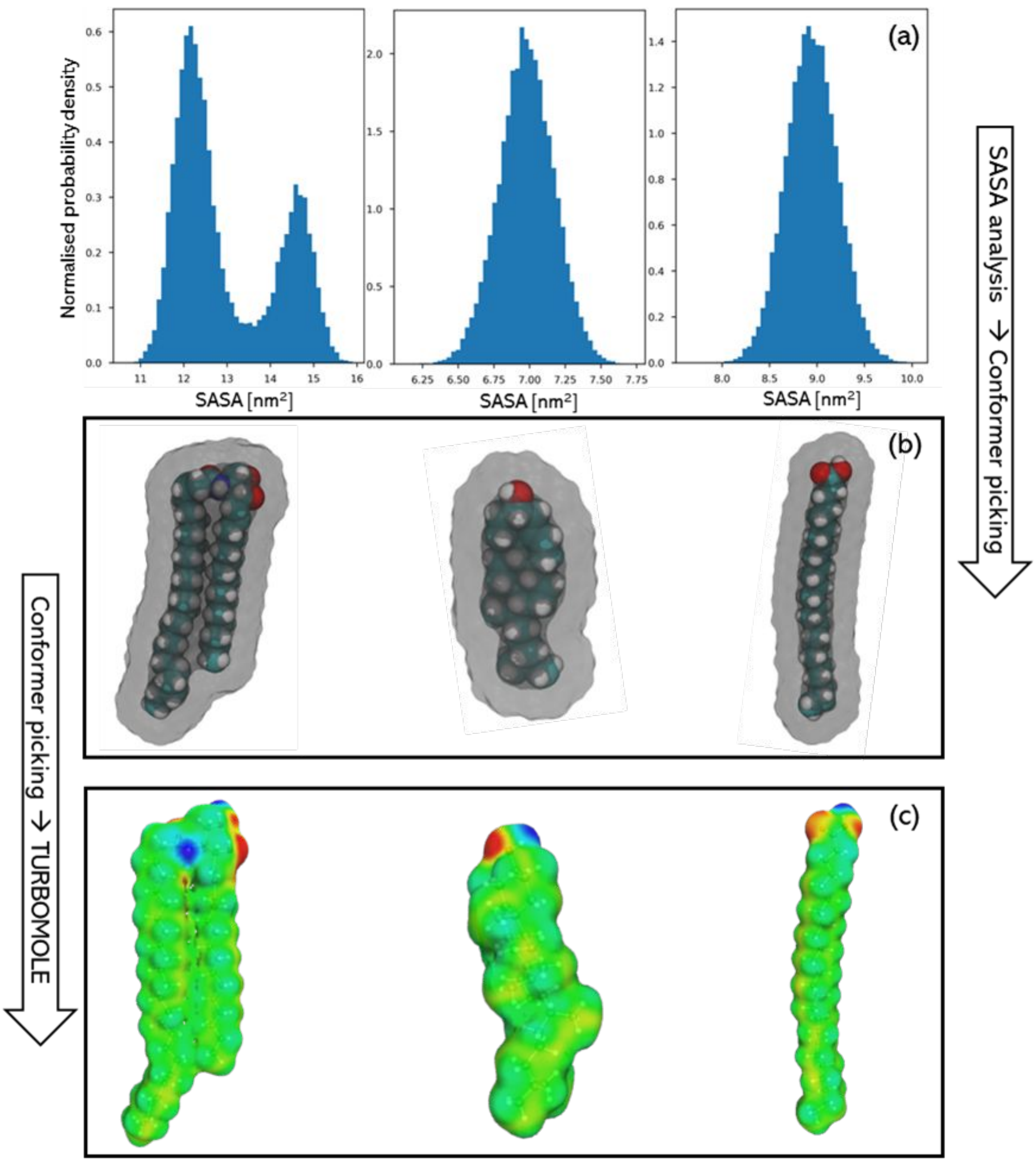

Figure S1 Example of lipid conformer search for a system. (a) After the simulation, the last 10 ns of run are analysed via gmx sasa. (b) A conformer with the average SASA is extracted from the corresponding snapshot of the given system. The grey surface is the representation of the SASA [illegible]

Number density and SASA distributions

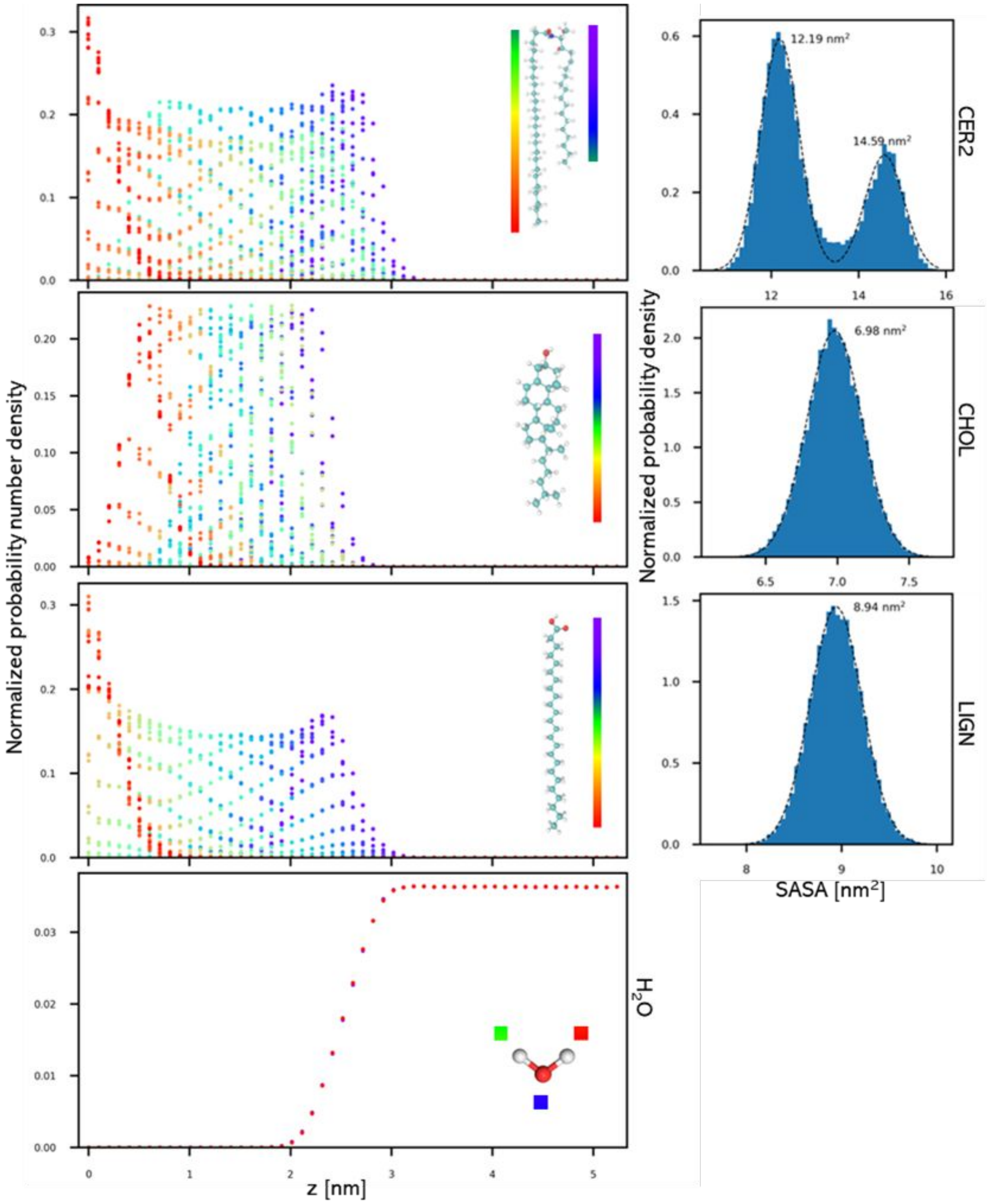

Figure S2 Left, top to bottom - Normalized number density for CER2, CHOL, LIGN, and water in HPm2. Right, top to bottom - SASA distribution for CER2, CHOL, and LIGN in HPm2.

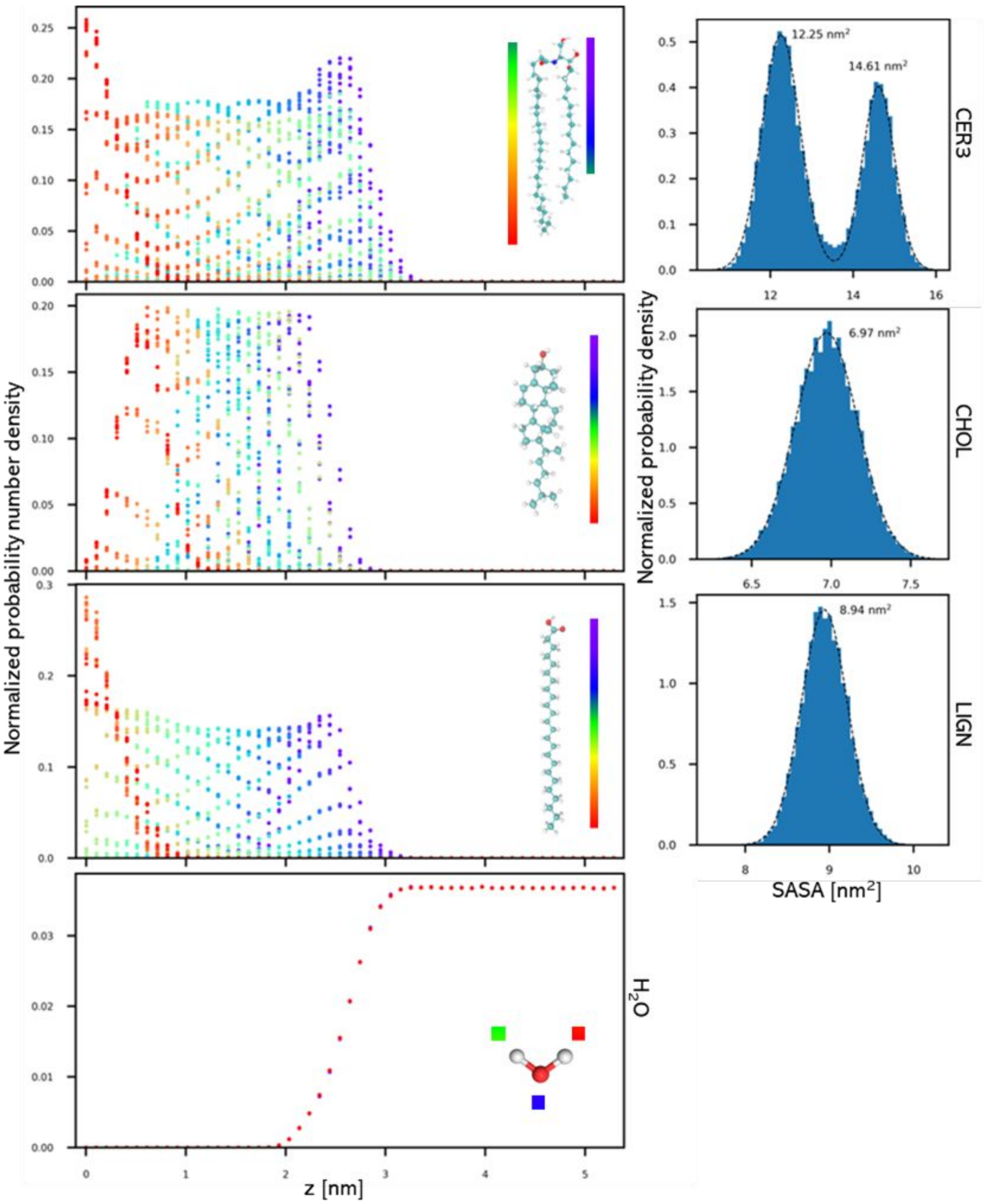

Figure S3 Left, top to bottom - Normalized number density for CER3, CHOL, LIGN, and water in HPm3. Right, top to bottom - SASA distribution for CER3, CHOL, and LIGN in HPm3.

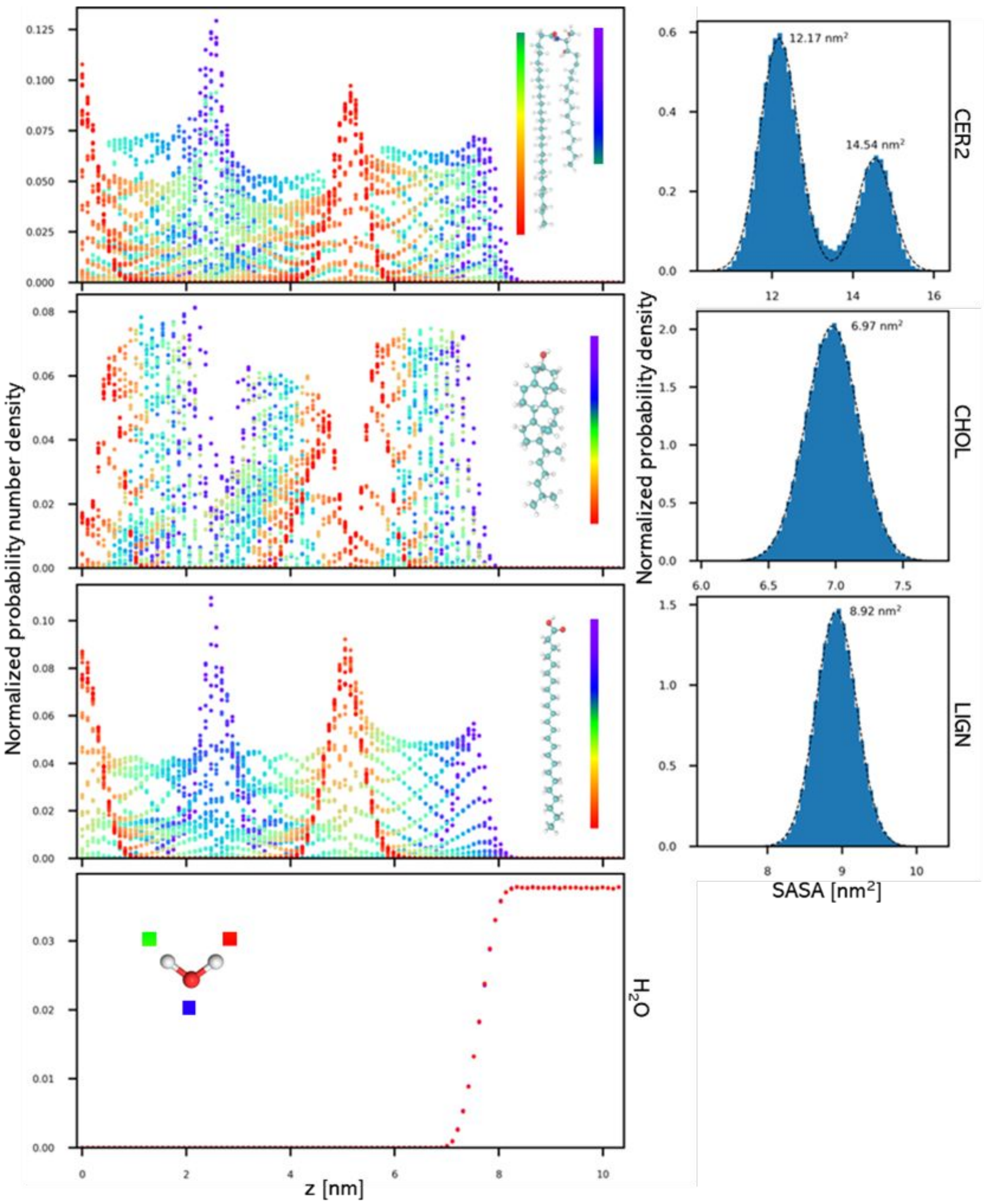

Figure S4 Left, top to bottom - Normalized number density for CER2, CHOL, LIGN, and water in HPm2t. Right, top to bottom - SASA distribution for CER2, CHOL, and LIGN in HPm2t.

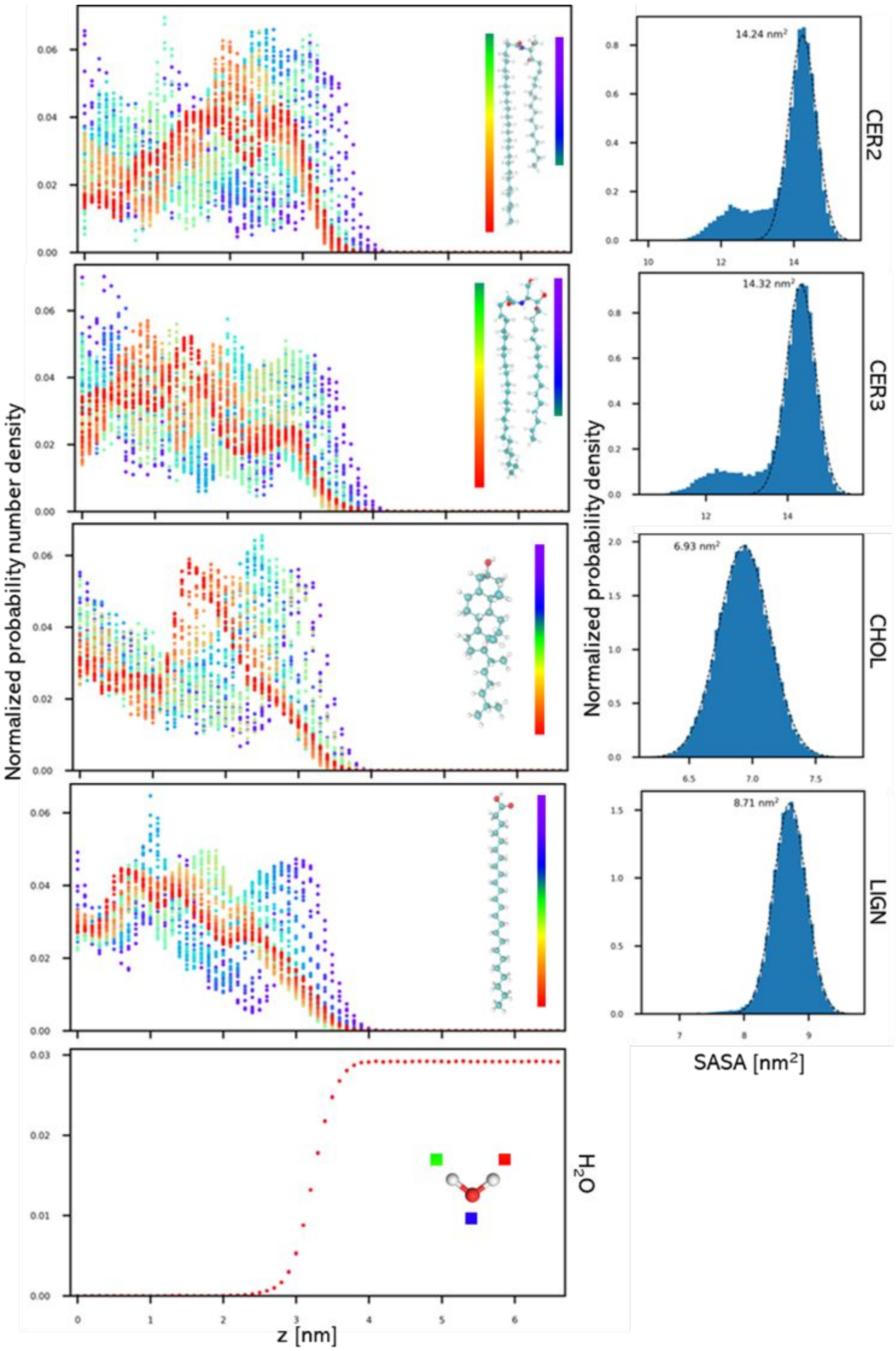

Figure S5 Left, top to bottom - Normalized number density distribution for CER2, CER3, CHOL, LIGN, and water in Amor. Right, top to bottom - SASA distribution for CER2, CER3, CHOL, and LIGN in Amor.

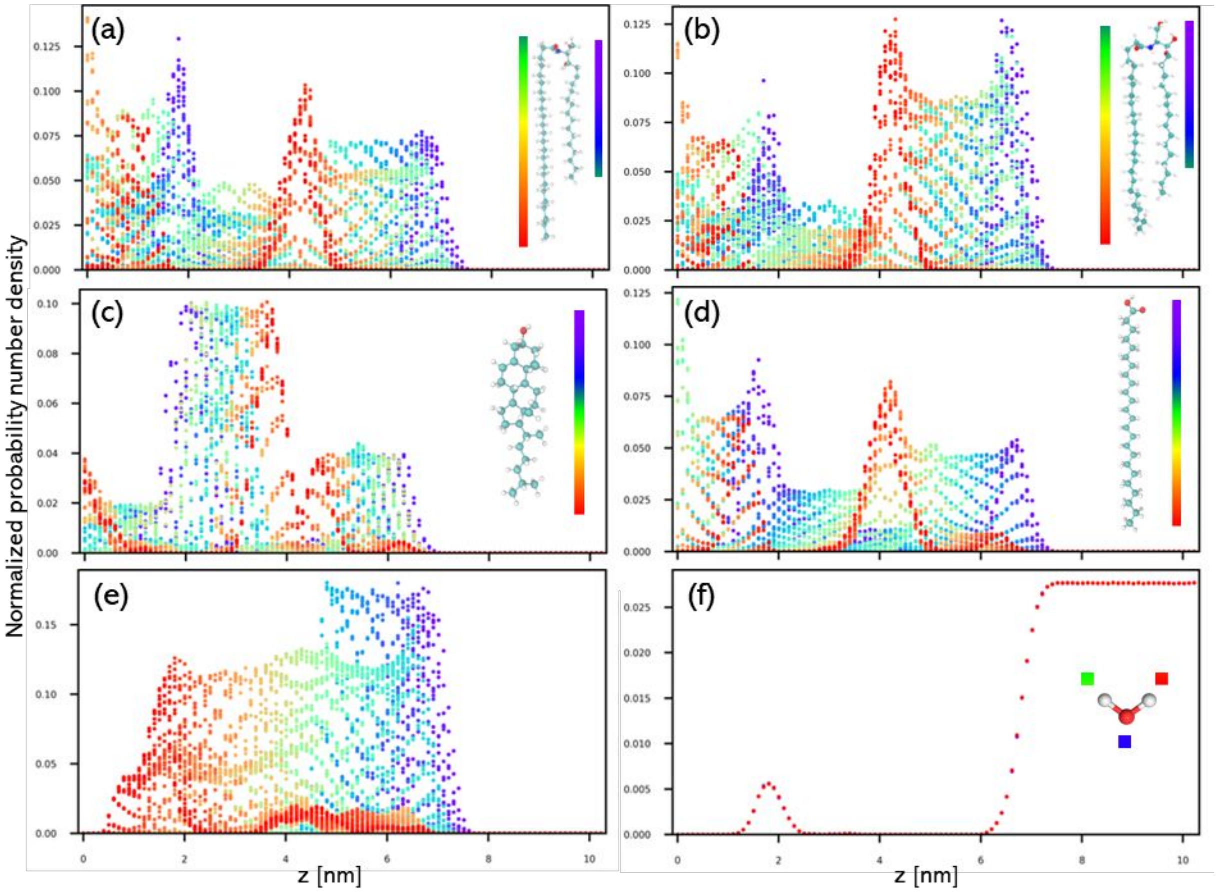

Figure S6 Normalized number density distribution for (a) CER2, (b) CER3, (c) CHOL, (d) LIGN, (e) CERO, and (f) water in LPP.

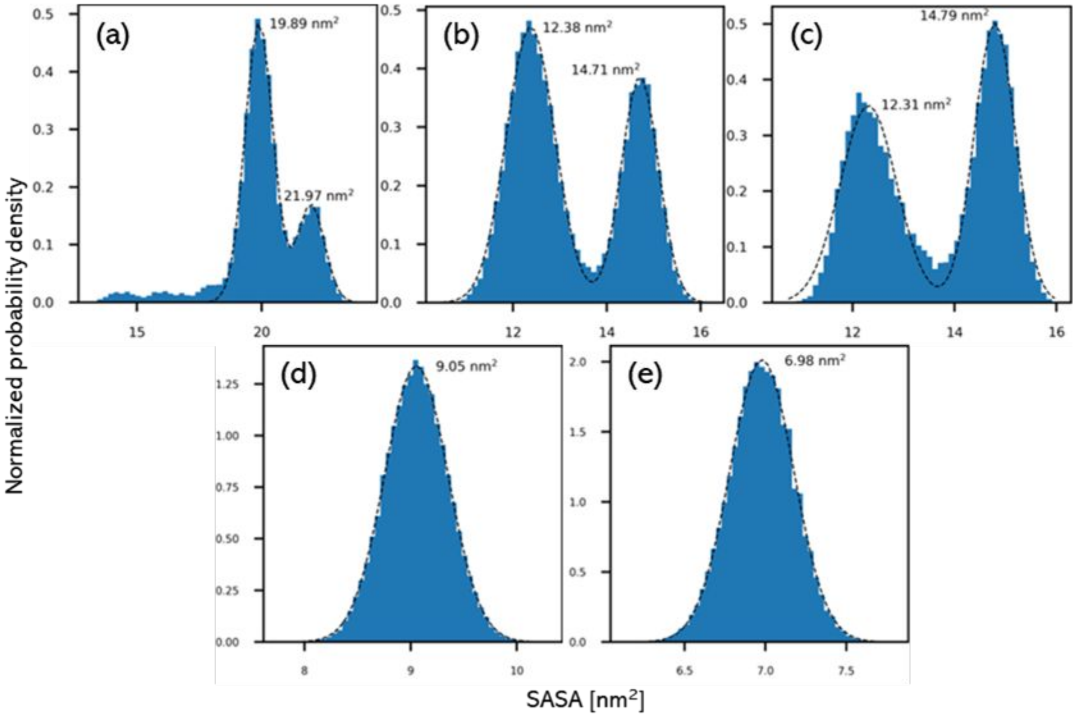

Figure S7 SASA distribution for (a) CERO, (b) CER2, (c) CER3, (d) LIGN, and (e) CHOL in LPP.

Distributions of SASA from figures S2 to S7 are fitted with one (CHOL and LIGNP) or two (CERs) gaussians. In a few cases, CERs distributions showed only one peak and have been therefore fitted with only one gaussian. The fit has been performed via python3 with the lmfit[1] package. The gaussian has three independent parameters for the fit, that is the mean μ, the standard deviation σ, and the amplitude A. For two peaks, the sum of the gaussians has the following form

$$\frac{A_1}{\sqrt{2\pi}\sigma_1}\exp\left[-\frac{(x-\mu_1)^2}{2\sigma_1^2}\right]+\frac{A_2}{\sqrt{2\pi}\sigma_2}\exp\left[-\frac{(x-\mu_2)^2}{2\sigma_2^2}\right] \tag{1}$$

Where the subscripts 1 and 2 indicate the two gaussians. For the one-gaussian fit, only the first term is considered ($A_2 = 0$). Table S1 summarizes all the fit values obtained. For the cases where an average is performed, reported as $\bar{\mu}$, the average is calculated by weighting the two averages μ_i for the corresponding amplitude A_i.

Table S1 Summary of all gaussian fit for the SASA of all the systems. All values are reported in [nm].

		CER2		CER3		CERO		LIGN	CHOL
**HPm2**	μ	12.19	14.59	-	-	-	-	6.98	8.94
	h	0.59	0.30	-	-	-	-	2.07	1.46
	σ	0.44	0.45	-	-	-	-	0.19	0.27
	A	0.66	0.33	-	-	-	-	1.00	1.00
	$\bar{\mu}$	13.00		-		-		-	-
**HPm3**	μ	-	-	12.25	14.61	-	-	6.97	8.94
	h	-	-	0.52	0.41	-	-	2.03	1.46
	σ	-	-	0.46	0.39	-	-	0.20	0.27

	A	-	-	0.60	0.39	-	-	1.00	1.00
	$\bar{\mu}$	-		13.19		-		-	-
**HPm2t**	μ	12.19	14.59	-	-	-	-	6.97	8.92
	h	0.59	0.30	-	-	-	-	2.03	1.46
	σ	0.44	0.45	-	-	-	-	0.20	0.27
	A	0.66	0.33	-	-	-	-	1.00	1.00
	$\bar{\mu}$	13.00		-		-		-	-
**Amor**	μ	14.24		14.32		-	-	6.93	8.71
	h	0.85		0.93		-	-	1.96	1.56
	σ	0.37		0.36		-	-	0.20	0.25
	A	0.79		0.83		-	-	1.00	0.99
	$\bar{\mu}$	-		-		-		-	-
**LPP**	μ	12.38	14.71	12.31	14.79	19.89	21.97	9.05	6.98
	h	0.47	0.39	0.35	0.50	0.49	0.17	1.34	2.01
	σ	0.52	0.40	0.55	0.40	0.57	0.54	0.30	0.20
	A	0.61	0.39	0.49	0.51	0.69	0.23	1.00	1.00
	$\bar{\mu}$	13.27		13.57		20.42		-	-

2. Partition coefficient calculation

As reported in the main text, the lipid/water partition coefficient K_{lip}^{ext} can be calculated[2] as

$$K_{lip}^{ext} = \frac{\sum_{i=1}^{n}\left[V(z_i)\cdot \exp\left(-\frac{\Delta G(z_i)}{RT}\right) - V(z_n)\cdot \exp\left(-\frac{\Delta G(z_n)}{RT}\right)\cdot \frac{x_w(z_i)}{x_w(z_n)}\right]}{V(z_n)\cdot \exp\left(-\frac{\Delta G(z_n)}{RT}\right)\cdot \frac{1}{x_w(z_n)}} \quad (2)$$

where n is the total number of slices of the system, $V(z_i)$ is the volume of the ith slice, $\Delta G(z_i)$ is the free energy at z_i, R is the universal gas constant, T is the absolute temperature, $n_w(z_i)$ is the number of water molecules contained in the ith slice, and $n_{w,tot}$ is the total number of water molecules in the system. Lastly, $x_w(z_i) = n_w(z_i)/n_{w,tot}$ with $n_w(z_i)$ being the number of water molecules contained in the ith slice and $n_{w,tot}$ being the total number of water molecules in the system. Thus, $x_w(z_i)$ is fraction of water molecules present in the slice *i*, and it's equal to the value at $z = z_i$ of the normalized probability distribution histogram for water.

K_{lip}^{ext} in Eq.(2) is an extensive quantity, meaning that its value depends on the system size, and has units of mol/mol. It can be converted[3, 4] to an intensive coefficient K_{lip}, with units of moles of solute per kg of lipid over moles of solute per liter of water [L/kg], by multiplying it for the ratio of the water phase volume V_w over the total lipid mass $m_{lip,tot}$

$$\frac{V_\mathrm{w}}{m_\mathrm{lip,tot}} = \frac{N_\mathrm{A} \cdot V(z_\mathrm{n})}{M_\mathrm{lip} \cdot \frac{n_\mathrm{lip}}{2}} \cdot \frac{1}{x_\mathrm{w}(z_\mathrm{n})} \tag{3}$$

where N_A is the Avogadro number, n_{lip} is the total number of lipids, and M_{lip} is the molecular weight of the lipids.

Since the simulations presented in this work have all rectangular boxes and the system is split along the *z* axis in slices of equal thickness, it follows that all the volumes $V(z_i)$ are independent from *i*, that is, they have the same value δV. Then, Eq.(2), when converted to [L/kg] via Eq.(3) and by exploiting the symmetry $V(z_i) = \delta V \;\forall\; i = 1,\ldots,n$, becomes

$$\begin{aligned}
K_\mathrm{lip} &= \frac{V_\mathrm{w}}{m_\mathrm{lip,tot}} \times K_\mathrm{lip}^\mathrm{ext} \\
&= \frac{N_\mathrm{A} \cdot V(z_\mathrm{n})}{M_\mathrm{lip} \cdot \frac{n_\mathrm{lip}}{2}} \frac{1}{x_\mathrm{w}(z_\mathrm{n})} \times\times \frac{\sum_{i=1}^{n} \left[V(z_\mathrm{i}) \cdot \exp\left(-\frac{\Delta G(z_\mathrm{i})}{RT}\right) - V(z_\mathrm{n}) \cdot \exp\left(-\frac{\Delta G(z_\mathrm{n})}{RT}\right) \cdot \frac{x_{[illegible]}}{x_{[illegible]}} \right.}{V(z_\mathrm{n}) \cdot \exp\left(-\frac{\Delta G(z_\mathrm{n})}{RT}\right) \cdot \frac{1}{x_\mathrm{w}(z_\mathrm{n})}} \\
&= \frac{N_\mathrm{A} \cdot \delta V}{M_\mathrm{lip} \cdot \frac{n_\mathrm{lip}}{2}} \times \frac{\sum_{i=1}^{n} \left[\delta V \cdot \exp\left(-\frac{\Delta G(z_\mathrm{i})}{RT}\right) - \delta V \cdot \exp\left(-\frac{\Delta G(z_\mathrm{n})}{RT}\right) \cdot \frac{x_\mathrm{w}(z_\mathrm{i})}{x_\mathrm{w}(z_\mathrm{n})} \right]}{\delta V \cdot \exp\left(-\frac{\Delta G(z_\mathrm{n})}{RT}\right)} \\
&= \frac{N_\mathrm{A} \cdot \delta V}{M_\mathrm{lip} \cdot \frac{n_\mathrm{lip}}{2}} \times \frac{\sum_{i=1}^{n} \left[\exp\left(-\frac{\Delta G(z_\mathrm{i})}{RT}\right) - \exp\left(-\frac{\Delta G(z_\mathrm{n})}{RT}\right) \cdot \frac{x_\mathrm{w}(z_\mathrm{i})}{x_\mathrm{w}(z_\mathrm{n})} \right]}{\exp\left(-\frac{\Delta G(z_\mathrm{n})}{RT}\right)}
\end{aligned} \tag{4}$$

$$= 2\frac{N_A \cdot \delta V}{M_{lip} \cdot n_{lip}} \times \sum_{i=1}^{n}\left[\exp\left(-\frac{\Delta G(z_i) - \Delta G(z_n)}{RT}\right) - \frac{x_w(z_i)}{x_w(z_n)}\right]$$

3. Reduced predicted log K_{lip} datasets

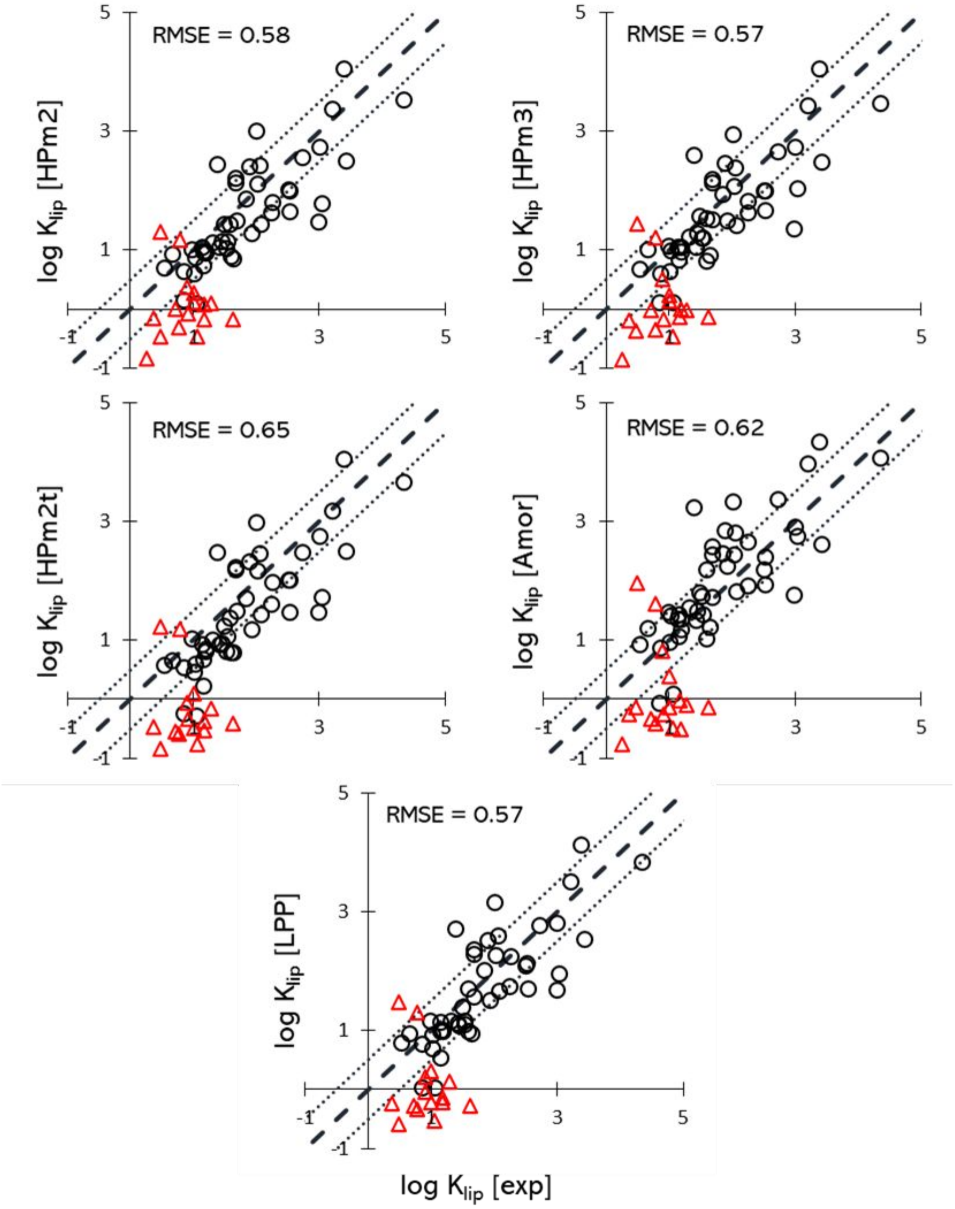

Figure S8 Predicted log K_{lip} plotted against the corresponding experimental values. The thick dashed line indicates perfect agreement, the two thin dotted lines highlight the ± 0.5 log point interval. Datasets are split into experimental log K_{ow} ≥ 1 (black open circles) and log K_{ow} < 1 (red open triangles). RMSE is calculated only for log K_{ow} ≥ 1.

Table S2 List of molecules with experimental log $K_{ow} < 1.0$, their corresponding molecular weight (MW), octanol/water partition coefficients log K_{ow}, experimental lipid/water partition coefficients log K_{lip}, and residues (RES), that is, the difference between the predicted and the experimental log

Molecule name	Experimental			Residuals				
	MW	log K_{ow}	log K_{lip}	HPm2	HPm3	HPm2t	Amor	LPP
p-Phenylenediamine	108.1	-0.30	1.05	-1.52	-1.51	-1.81	-1.53	-1.58
Caffeine	194.2	-0.07	0.48	-0.95	-0.85	-1.31	-0.61	-1.06
Theophylline	180.2	-0.02	0.26	-1.10	-1.12	-1.63	-1.02	-1.34
p-Aminophenol	109.1	0.04	0.78	-1.09	-1.12	-1.37	-1.19	-1.12
Thioglycolic acid	92.1	0.09	0.37	-0.52	-0.56	-0.84	-0.62	-0.61
HC Red No. 3	197.2	0.11	1.00	-0.89	-0.86	-1.49	-1.14	-1.22
Methyl methanesulfonate	110.1	0.20	1.62	-1.79	-1.75	-2.03	-1.75	-1.89
2,5-Diaminotoluene	122.2	0.34	1.17	-1.34	-1.31	-1.55	-1.20	-1.31
2-Nitro-1,4-phenylenediamine	153.1	0.53	0.71	-0.71	-0.72	-1.25	-1.05	-0.99
Diethylene glycol monobutyl ether	162.2	0.56	0.48	0.83	0.95	0.75	1.48	0.99
Hydroquinone	110.1	0.59	0.91	-0.99	-1.08	-1.24	-1.16	-0.95
4-Chlorobutyric acid	122.6	0.77	1.00	-0.72	-0.76	-0.91	-0.61	-0.69
Resorcinol	110.1	0.80	1.28	-1.18	-1.30	-1.43	-1.37	-1.14
Diethyl maleate	172.2	0.82	0.79	0.37	0.42	0.39	0.82	0.51
4-Nitro-1,2-phenylenediamine	153.1	0.88	1.18	-1.10	-1.17	-1.70	-1.69	-1.39
Aldosterone	360.4	0.96	0.90	-0.53	-0.40	-0.97	-0.08	-0.69

K_{lip} values. Cells colour code: $|RES| > 1$ (red), $1 \geq |RES| \geq 0.5$ (yellow), and $|RES| < 0.5$ (green).

4. Fit of experimental log K_{lip} data

As reported in the main text, the experimental SC lipid/water partition coefficients log K_{lip} have been compiled together. The data comes from Wang *et al.*[5] (and references therein) and Ellison *et al.*[6] and is fitted against the following linear relationship

$$\log K_{lip} = \beta \cdot \log K_{ow} \quad (5)$$

Where K_{ow} is the octanol/water partition coefficient of the solute. The log K_{ow} values are taken from the United States Environmental Protection Agency (EPA)[7] dashboard. The best fit results are $\beta = 0.74$ with RMSE = 0.46 and R = 0.84. The dataset is plotted against the best fitted linear relationship in Figure S9.

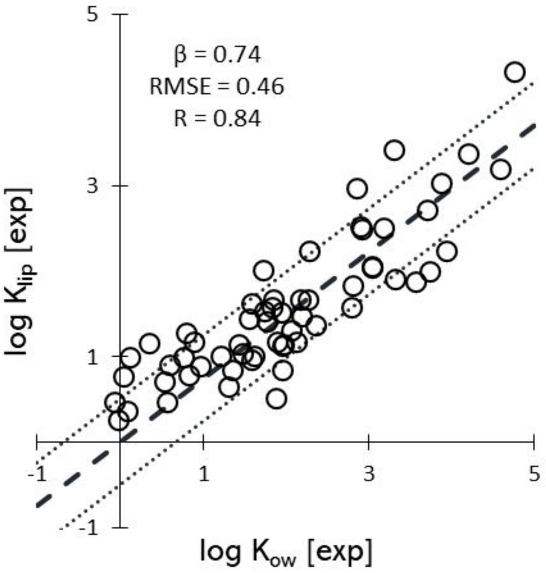

Figure S9 Experimental dataset plotted against the best fit result (dashed line). The dotted lines highlight the ±0.5 log points interval.

Experimental values are reported in the additional Supplementary Information sheet. Five molecules have been removed from the dataset, namely 4-bromophenyl isocyanate, flufenamic acid, hydrocortisone octanoate, methyl methanesulfonate, and p-phenylenediamine. The first two have been removed because their log K_{ow} is greater than 5, and this could lead to imprecise experimental measurements[8]. The last three have been removed as large outliers (residuals > 1). Their inclusion decreases the correlation coefficient R but doesn't affect the linear coefficient (β = 0.75, RMSE = 0.54, R = 0.77).

5. Analysis of predicted log K_{lip} as a function of log K_{ow}

Residuals analysis

Figure S10 and Figure S11 show the residuals of the predictions plotted against the residuals of the log K_{ow}, fitted against a line without intercept. In the main paper, the predicted log K_{lip} values have been fit to the linear relationship in Eq.(5) both for the case of experimental and predicted log K_{ow}. The results consist in a series of angular coefficients β for each system. In the following, angular coefficients obtained from fits against experimental log K_{ow} (log K_{ow}^{exp}) are denoted as β_{exp}, while those obtained from fits against COSMOtherm-predicted log K_{ow} (log K_{ow}^{pre}) are denoted as β_{pre}. The residuals (RES) are defined as

- difference between predicted (pre) and experimental (exp) log K_{lip} (blue open circles, first columns in Figure S10 and Figure S11)

$$\log K_{lip}^{pre} - \log K_{lip}^{exp} \quad (6)$$

- difference between predicted log K_{lip} and fit against experimental log K_{ow} (orange open squares, second columns in Figure S10 and Figure S11)

$$\log K_{lip}^{pre} - \beta_{exp} \cdot \log K_{ow}^{exp} \quad (7)$$

- difference between predicted log K_{lip} and fit against predicted log K_{ow} (green open triangles, third columns in Figure S10 and Figure S11)

$$\log K_{lip}^{pre} - \beta_{pre} \cdot \log K_{ow}^{pre} \quad (8)$$

- difference between predicted and experimental log K_{ow} (the abscissa in all subplots in Figure S10 and Figure S11)

$$\log K_{ow}^{pre} - \log K_{ow}^{exp} \quad (9)$$

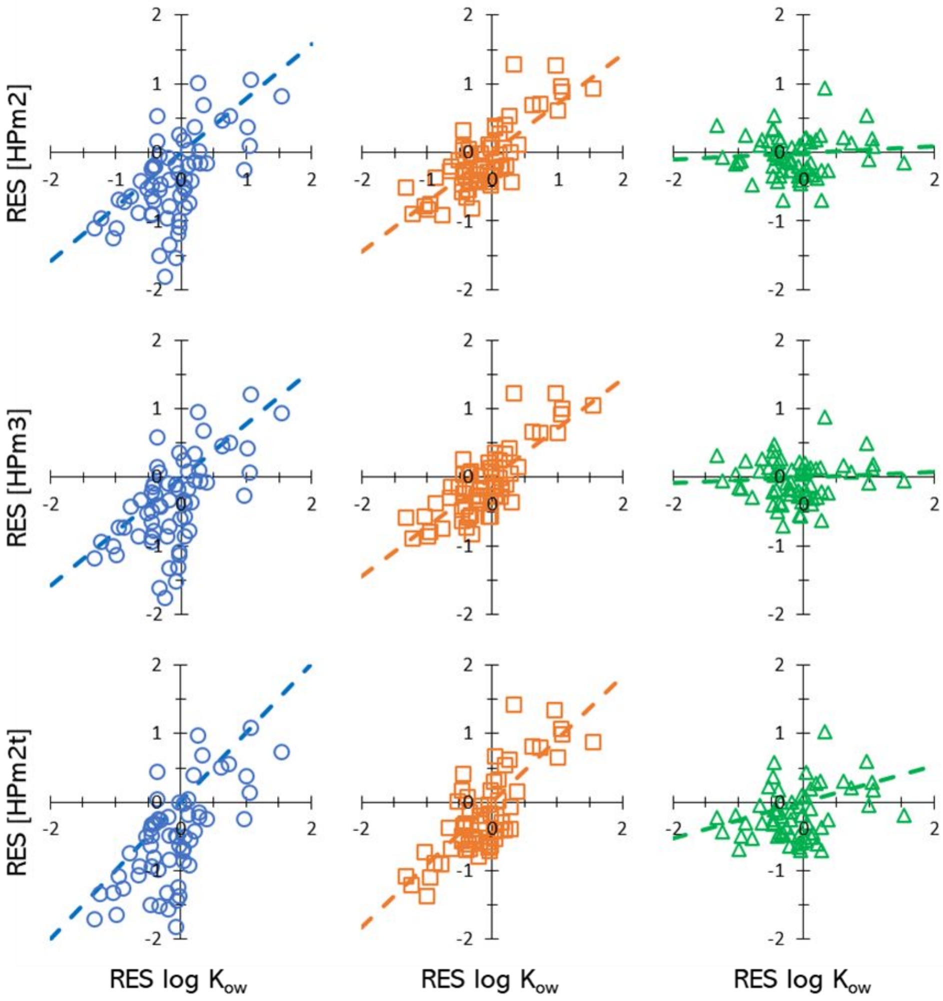

Figure S10 Residuals for predicted vs. experimental log K_{lip} (as in Eq.(6), first column, blue open circles), for predicted vs. fitted log K_{lip} to experimental log K_{ow} (as in Eq.(7), second column, orange open squares), and for predicted vs. fitted log K_{lip} to predicted log K_{ow} (as in Eq.(8), third column, green open triangles), as a function of the residual between predicted and experimental log K_{ow} (as in Eq.(9)) for the hairpin systems (HPm2, HPm3, and HPm2t).

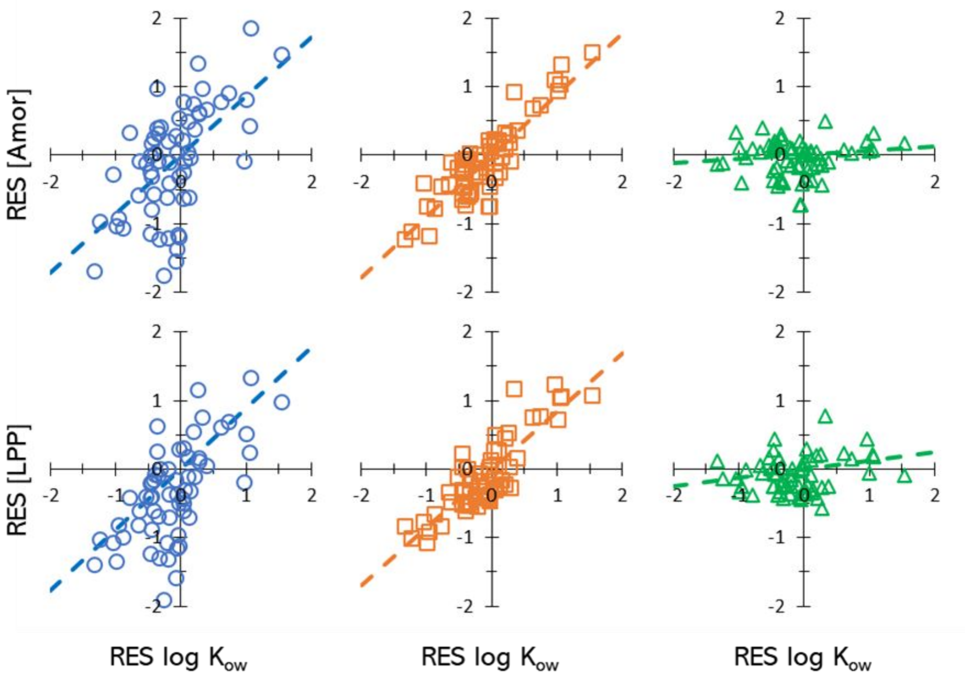

Figure S11 Residuals for predicted vs. experimental log K_{lip} (as in Eq.(6), first column, blue open circles), for predicted vs. fitted log K_{lip} to experimental log K_{ow} (as in Eq.(7), second column, orange open squares), and for predicted vs. fitted log K_{lip} to predicted log K_{ow} (as in Eq.(8), third column, green open triangles), as a function of the residual between predicted and experimental log K_{ow} (as in Eq.(9)) for Amor and LPP systems.

Summary of fit results

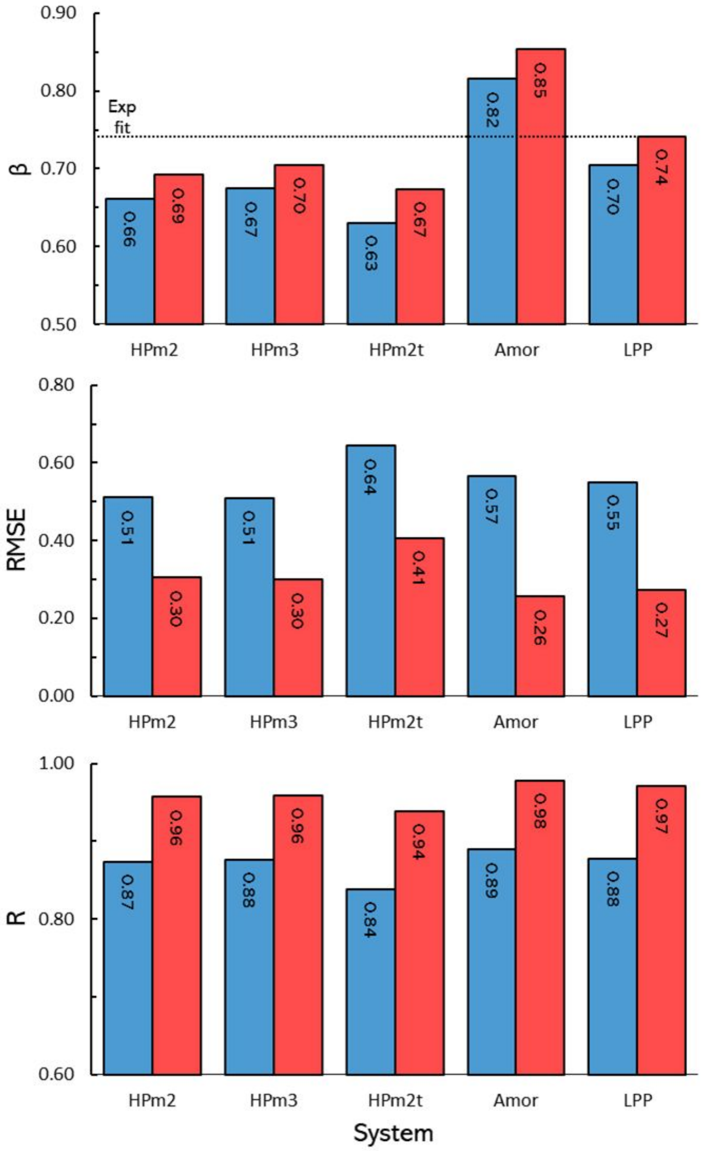

Figure S12 Summary of all the results for the linear fit between predicted log K_{lip} and experimental (blue) and predicted (red) log K[illegible]

6. References

(1) Newville, M.; Stensitzki, T.; Allen, D. B.; Rawlik, M.; Ingargiola, A.; Nelson, A. LMFIT: Non-linear least-square minimization and curve-fitting for Python. *Astrophysics Source Code Library* **2016**, ascl: 1606.1014.

(2) Klamt, A.; Huniar, U.; Spycher, S.; Keldenich, J. r. COSMOmic: a mechanistic approach to the calculation of membrane− water partition coefficients and internal distributions within membranes and micelles. *The Journal of Physical Chemistry B* **2008**, *112* (38), 12148-12157.

(3) Jakobtorweihen, S.; Ingram, T.; Smirnova, I. Combination of COSMOmic and molecular dynamics simulations for the calculation of membrane–water partition coefficients. *Journal of computational chemistry* **2013**, *34* (15), 1332-1340.

(4) Jakobtorweihen, S.; Zuniga, A. C.; Ingram, T.; Gerlach, T.; Keil, F.; Smirnova, I. Predicting solute partitioning in lipid bilayers: Free energies and partition coefficients from molecular dynamics simulations and COSMOmic. *The Journal of Chemical Physics* **2014**, *141* (4), 07B622_621.

(5) Wang, L.; Chen, L.; Lian, G.; Han, L. Determination of partition and binding properties of solutes to stratum corneum. *Int J Pharm* **2010**, *398* (1-2), 114-122. DOI: 10.1016/j.ijpharm.2010.07.035.

(6) Ellison, C. A.; Tankersley, K. O.; Obringer, C. M.; Carr, G. J.; Manwaring, J.; Rothe, H.; Duplan, H.; Géniès, C.; Grégoire, S.; Hewitt, N. J. Partition coefficient and diffusion coefficient determinations of 50 compounds in human intact skin, isolated skin layers and isolated stratum corneum lipids. *Toxicology in Vitro* **2020**, *69*, 104990.

(7) Williams, A. J.; Grulke, C. M.; Edwards, J.; McEachran, A. D.; Mansouri, K.; Baker, N. C.; Patlewicz, G.; Shah, I.; Wambaugh, J. F.; Judson, R. S. The CompTox Chemistry Dashboard: a community data resource for environmental chemistry. *Journal of cheminformatics* **2017**, *9* (1), 1-27.

(8) Nitsche, J. M.; Wang, T.-F.; Kasting, G. B. A two-phase analysis of solute partitioning into the stratum corneum. *Journal of pharmaceutical sciences* **2006**, *95* (3), 649-666.
